# Youth peer-based mental health programmes and supports in low- and middle-income countries: rapid review

**DOI:** 10.1192/bjo.2026.11030

**Published:** 2026-05-06

**Authors:** Joseph H. Puyat, Mana Mohebbian, Erin Kwak, Jaime A. Manalo, Mehar Mago, Mark Andre Blanco, John Ismael J. Medina, Ursula Ellis, Llewelyn Issa B. Dela Cruz, Carolina Uno-Rayco, Mendiola Teng-Calleja, Carla T. Hilario, Raymond W. Lam, Rosemin Kassam

**Affiliations:** School of Population and Public Health, Faculty of Medicine, The University of British Columbia, Canada; Centre for Advancing Health Outcomes, Providence Health Care, Vancouver, Canada; Socioeconomics Division, Philippine Rice Research Institute, Science City of Muñoz, Philippines; Neuroscience Programme, The University of British Columbia, Canada; College of Public Health, University of the Philippines Manila, Philippines; SHS Health Allied, University of Santo Tomas, Manila, Metro Manila, Philippines; Woodward Library, The University of British Columbia, Canada; Philippine Mental Health Association, Quezon City, Philippines; Department of Psychology, Ateneo de Manila University, Philippines; School of Nursing, The University of British Columbia Okanagan, Canada; Department of Psychiatry, Faculty of Medicine, The University of British Columbia, Canada

**Keywords:** Systematic review, low- and middle-income countries, mental health services, peer support, youth

## Abstract

**Background:**

Youth in low- and middle-income countries (LMICs) bear a disproportionate burden of mental health conditions, alongside low health-seeking behaviours and limited access to services. These gaps underscore the need for accessible strategies such as youth peer-based mental health programmes and supports (Y-PBMHPS).

**Aims:**

To examine whether Y-PBMHPS can help address the mental health needs of LMIC youth.

**Method:**

We conducted a rapid review of peer-reviewed literature, searching Medline, PsycINFO, CINAHL, CAB Global Health, Science Citation Index and Social Sciences Citation Index for studies of Y-PBMHPS in LMICs published in English between 1 January 2002 and 19 September 2025. Two review authors performed title/abstract screening and full-text review. Study quality was assessed by one review author using Joanna Briggs Institute critical appraisal tools. The primary outcome was change in mental health status, expressed in standardised difference units.

**Results:**

Of 6105 unique records identified, 329 studies were reviewed in full and 34 were included. All studies were conducted in Asia or Africa; 17 were quantitative studies (including randomised controlled trials), 9 were qualitative studies and 8 used quantitative designs with qualitative findings. Y-PBMHPS included counselling, psychotherapy, psychoeducation and self-help groups, with peers acting as leaders, facilitators, educators or service providers. Quantitative studies most frequently assessed anxiety and depression, reporting negligible to moderate effects. Qualitative findings indicated good fidelity, adherence and acceptability, alongside some feasibility challenges.

**Conclusions:**

Y-PBMHPS can broaden youth mental health support and services in LMICs. Clearer guidelines on peer selection, training and supervision and further research in other LMICs, including cost-effectiveness evaluations, would strengthen the evidence base.

## Mental health burden in youth

Approximately 20% of the world’s children and adolescents live with various mental health conditions, with anxiety and depression being the most prevalent^1,2^ and suicide as a leading cause of death.^3^ Given that almost 90% of adolescents in the world live in low- and middle-income countries (LMICs), youth mental health in these settings requires global priority.^4^ This population is disproportionately exposed to social determinants, such as poverty, instability and limited access to mental health services, compounding the risk of mental illness.^5^ Many mental health conditions experienced by adults have early onsets, with symptoms typically manifesting during childhood and adolescence.^6^ Although the burden of mental health conditions is high among youth, help-seeking is relatively low because of various factors, including scarce mental health resources, stigma, negative beliefs toward mental health services and treatment, poor mental health literacy, and perceived need for self-sufficiency and autonomy.^7^ This low rate of help-seeking behaviour during adolescence is a concern, as this can lead to poor mental and physical health in adulthood.^8,9^

## Youth peer-based mental health programmes and supports

A promising yet understudied approach to addressing the gap in supports and services for children and youth is the use of youth peer-based mental health programmes and supports (Y-PBMHPS), in which young people with shared lived experience are trained to provide structured support, information or linkage to care to other youth within community or institutional settings. To date, research on the development and implementation of Y-PBMHPS for youth has been largely restricted to high-income countries (HICs).^10^ In these settings, peer-delivered interventions show evidence of beneficial effects and equivalence with peer and professionally delivered interventions.^11,12^ One obvious advantage of Y-PBMHPS is that they are not heavily dependent on specialist mental health services, which tend to be limited, expensive or associated with long wait times.^13^ Y-PBMHPS, especially those based in schools with large enrolments, have access to a large number of peer supporters, resulting in accessible, lower-cost services.^14,15^ Y-PBMHPS also have the added advantage of dually benefiting the peer supporters and the individuals they support.^16^ Their potential utility is further reinforced by the central role of peer influence in shaping young people’s behaviours and help-seeking.^17^

Conceptually, Y-PBMHPS have the potential to address persistent barriers to mental healthcare access in LMICs by leveraging peers as trusted, low-pressure liaisons who can extend support, information and linkage to care within community settings.^18^ If effective, such programmes could expand service coverage, reduce treatment gaps and support more equitable task-shared models of care in resource-constrained health systems.^19,20^ Conversely, limited effectiveness or poor feasibility would have important implications for policy prioritisation and the allocation of scarce mental health resources. Their successful implementation is also likely to be shaped by contextual factors common in LMICs, including workforce capacity, training and supervision structures, cultural acceptability, stigma and long-term sustainability.

Building on these considerations, we conducted a rapid review of Y-PBMHPS implemented or developed in LMICs to inform both evidence and decision-making in resource-constrained settings. Specifically, this review sought to (a) characterise the types of Y-PBMHPS reported in LMICs and (b) examine the available evidence on their feasibility and effectiveness in improving mental health outcomes among youth in these contexts.

## Method

This review was conducted following the Preferred Reporting Items for Systematic Reviews and Meta-Analyses (PRISMA) guidelines (Supplementary Table 1) and the Joanna Briggs Institute (JBI) manual for evidence synthesis.^21^ The review protocol was retrospectively registered at PROSPERO (identifier CRD42022352576) on 20 August 2022, and made public after execution of the search strategy but before starting title/abstract screening.

In this review, we defined Y-PBMHPS as any support system led, facilitated or provided by people who share common experience or characteristics with the person receiving the support. We characterised peers as those belonging to the same age group and/or those with lived experience of mental health challenges or illness. We also adopted an expanded age range of 15–30 years to characterise youth, in recognition of the cultural and jurisdictional variations surrounding the definition of youth.^22,23^ We acknowledge that peers may share additional specific characteristics (e.g. health status, bereavement or other lived contexts) beyond age or shared experience of mental illness, which may also shape the nature and mechanisms of support.

### Criteria for eligible studies

The full inclusion/exclusion criteria are included as Supplementary Material (Supplementary Table 2). We considered for inclusion all peer-reviewed studies that investigated the effectiveness of Y-PBMHPS implemented in schools or in communities in LMICs. The interventions could include peer-based supports, programmes or services that were facilitated or led by youth with or without lived experience of mental illness or psychological distress. Additionally, the intervention could be aimed at specific subpopulations, such as young mothers and young people living with chronic conditions (e.g. HIV). The interventions must have a clearly defined group of clients other than the peer leaders, peer supporters or peer facilitators involved in service delivery. We considered all types of service modes of delivery, including those that were administered in person, through mobile devices or through computers.

To be included for full review, quantitative studies must be about investigations of the impact of an intervention on mental health as a primary or secondary outcome. These outcomes could be in the form of reduction in the incidence, prevalence and severity of mental illness (e.g. anxiety, depression, suicidal ideation), or improvement in psychosocial outcomes, mental health-related knowledge, attitudes and health-seeking behaviour. Qualitative studies were included if they explored youth’s experiences with the intervention, or youth’s perspectives about the intervention’s importance, feasibility, acceptability and accessibility.

We excluded studies that did not investigate an intervention, mental health service programme or supports (wrong study design). Studies that investigated interventions with the following characteristics were also excluded: not conducted in LMICs as classified by the World Bank;^24^ not intended for youth as the target client (wrong population); exclusively focused on psychiatric medications or mental health services that required mental health professionals (wrong intervention); and no reported outcomes relevant to mental health or psychosocial outcomes (wrong outcome). Protocols for an intervention and reviews or syntheses were excluded, as well as non-peer-reviewed materials such as books or book chapters, letters to the editor, commentaries, theses and dissertations. Lastly, non-English studies were excluded because of resource constraints for translation, and we acknowledge this as a potential source of language bias.

### Search strategy

Medline (Ovid), PsycINFO (EBSCO), CINAHL, CAB Global Health (CAB Direct), Science Citation Index and Social Sciences Citation Index (Web of Science) were searched for quasi-experimental studies, randomised trials and qualitative studies published in English from January 2002 to July 2022, with an updated search to include September 2025. We used search terms that included any relevant clusters of terms related to peer-facilitated interventions, such as ‘peer-led’, ‘peer-based’ and ‘peer-assisted’. Keywords for relevant outcomes included ‘mental health literacy’ as well as major mental illnesses such as ‘depression’, ‘anxiety’ and ‘substance use’ (Supplementary Material). The initial search strategy was executed by a review author (M. Mohebbian) on 29 July 2022, and updated by another review author (U.E.) on 19 September 2025.

### Screening and quality assessment

**Fig. 1:**
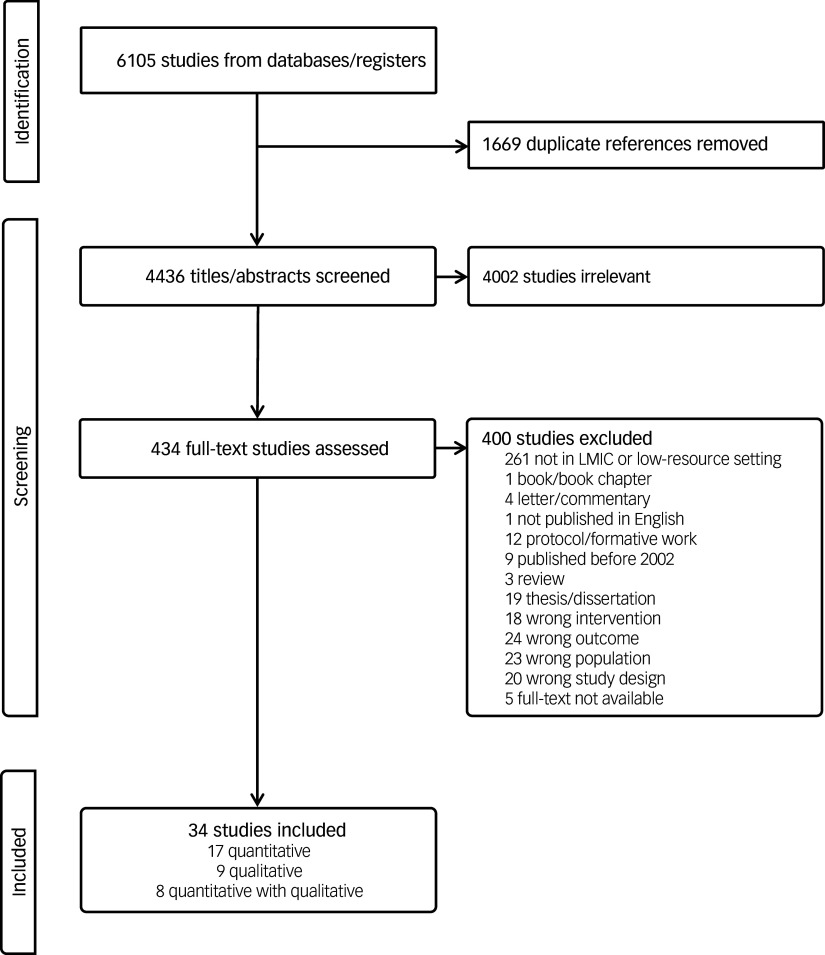
Preferred Reporting Items for Systematic reviews and Meta-Analyses flowchart. LMIC, low- and middle-income country.

Titles/abstracts were screened independently by two review authors (J.I.M., M. Mohebbian, J.A.M., E.K., M. Mago or J.H.P.). Conflicts in title/abstract screening were resolved by another review author (J.H.P., M. Mohebbian or E.K.). Full review of potentially eligible studies was also conducted by two review authors (J.A.M., M. Mohebbian, E.K., M. Mago, J.H.P., J.I.M. or M.A.B.). Conflicts during full-text review were resolved by consensus. All excluded studies during full review were reviewed by a review author (J.H.P.) and sent for full review when deemed potentially relevant. Some studies were excluded for multiple reasons, although only one reason for exclusion was reported (Fig. 1). We used the Covidence^25^ platform for screening and full-text review.

We used the following JBI tools to assess the quality of included studies: Checklist for Randomised Controlled Trials,^26^ Checklist for Quasi-Experimental Studies or Non-Randomised Experimental Studies^27^ and Checklist for Qualitative Research.^28^ One review author performed the primary quality assessment (J.A.M., M.A.B., M.M.1 or E.K.), followed by an independent review (M. Mohebbian, M.A.B., J.H.P. or M. Mago). Because JBI does not prescribe a numerical scoring system or thresholds for classifying study quality, a structured scoring approach was developed for this review to ensure transparency and consistency across designs.

Each JBI checklist item was scored as 0 = No, 1 = Unclear, 2 = Yes, and items were grouped into conceptually coherent domains that reflect key aspects of methodological rigour. For randomised controlled trials (RCTs), domains included (A) randomisation and allocation, (B) blinding, (C) outcome measurement and follow-up, and (D) analysis and reporting. For quasi-experimental studies, domains captured (A) cause and effect, (B) comparability, (C) outcome measurement and (D) statistical analysis. For qualitative studies, domains addressed (A) congruity, (B) methodology/methods, (C) representation and ethics, and (D) interpretation/conclusions.

Domain scores were summed and converted to percentages, and then multiplied by prespecified domain weights to reflect the relative importance of each methodological construct and to prevent domains with more items from dominating the overall score. Weighted domain scores were combined to produce an overall score (0–100%) that was then used to inform the overall assessment of methodological quality as high (≥75%), moderate (50–74%) or low (<50%).

### Data extraction and synthesis

Data extraction was initiated on 11 January 2023, and updated on 1 November 2025 by one review author (M. Mohebbian, J.A.M., J.I.M. or E.K.) and independently verified by another review author (J.H.P., M. Mohebbian or M. Mago). For each included study, we extracted information on study design, country where the study was conducted, number and characteristics of study participants, intervention setting, nature and characteristics of the intervention, study outcome and key findings (Supplementary Tables 3 and 4). We also extracted additional contextual descriptors to indicate when studies involved peers who shared specific characteristics beyond age and lived experience of mental illness (i.e. health status, bereavement or other lived contexts), to support cautious interpretation of comparability and transferability across different Y-PBMHPS. Where available, relevant outcomes and key findings from both the quantitative and qualitative components of the study were extracted.

In addition, where reported, we extracted quantitative information required to estimate standardised effect sizes, including group means and standard deviations, and sample sizes, to enable calculation of standardised mean differences (Cohen’s *d*).

Study findings were grouped by intervention type, study design and mental health outcome, organised according to DSM-V characteristics.^29^ No meta-analysis was performed due to the heterogeneity of interventions and populations investigated in the included studies. However, we followed guidelines from the Synthesis Without Meta-Analysis ^30^ to summarise review findings.

## Results

Our search strategy yielded 6105 titles and abstracts, of which 4436 were unique and 4002 were deemed irrelevant. Of the 434 potentially relevant articles, 5 were irretrievable despite correspondence with the authors. Full-text review of the 329 articles resulted in 34 eligible studies (Fig. 1).

**Fig. 2:**
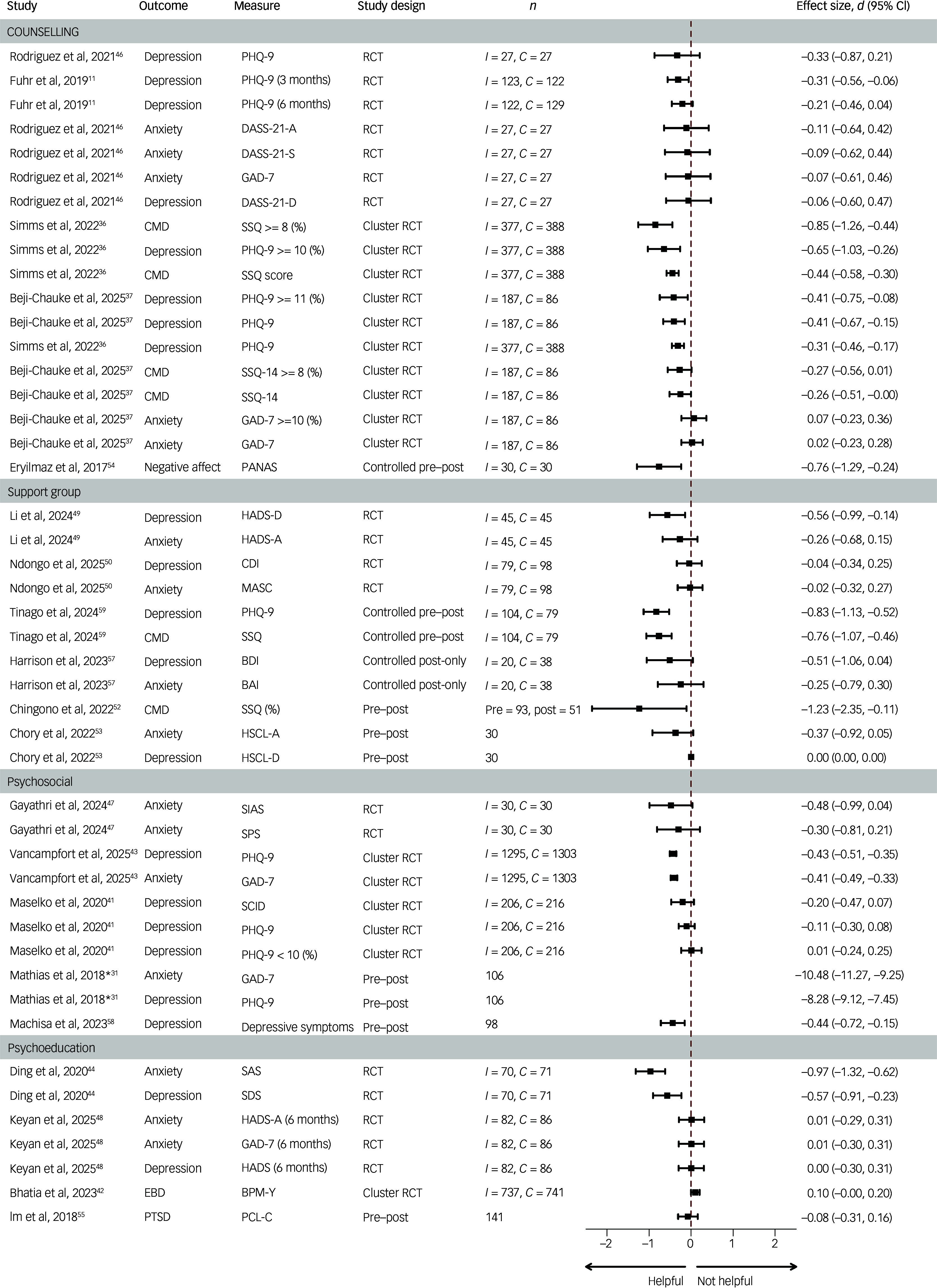
Effect sizes of selected youth peer-based mental health programmes and supports in low- and middle-income countries. *Effect size not plotted. *I* indicates the number of participants analysed in the intervention group; *C* indicates the number of participants analysed in the control group. BAI, Beck Anxiety Inventory; BDI, Beck Depression Inventory; BPM-Y, Brief Problem Monitor-Youth; CDI, Children’s Depression Inventory; CMD, Common Mental Disorders; DASS-21, Depression Anxiety Stress Scale, 21 items; DSS, Depresion Symptom Score; EBD, Emotional and Behavioral Difficulties; GAD-7, Generalised Anxiety Disorder, 7 items; HADS, Hospital Anxiety and Depression Scale; HSCL-A, Hopkins Symptom Checklist-Anxiety; HSCL-D, Hopkins Symptom Checklist-Depression; MASC, Multidimensional Anxiety Scale for Children; PANAS, Positive and Negative Affect Schedule; PCL-C, PTSD Check List-Civilian Version; PTSD, post-traumatic stress disorder; RCT, randomised controlled trial; SAS, Self-rating Anxiety Scale; SCID, Structured Clinical Interview for DSM Disorders; SDS, Self-Rating Depression Scale, 20 items; SIAS, Social Interaction Anxiety Scale; SPS, Social Phobia Scale; SSQ, Shona Symptom Questionnaire, 14 items.

Of the 34 included studies, 3 were based on the same peer-facilitated psychoeducation programme,^31–33^ 4 reported on the same peer-delivered counselling and support programmes,^34–37^ and 2 reported on the same screening and brief intervention programme.^38,39^ Because these nine studies reported on different aspects of the same intervention, we assessed them as separate studies. Of note, 23 of the included studies (over half) were published within the past 5 years (2020 onward), indicating a recent and growing evidence base in this area. Depression (*n* = 17) and anxiety (*n* = 11) were the most commonly reported outcomes (Supplementary Table 4 and Fig. 2).

Fifteen of the included articles were randomised studies, consisting of six cluster RCTs,^36,37,40–43^ eight traditional RCTs^11,44–50^ and one study described by the authors as quasi-experimental but assessed in this review as a traditional RCT because of the presence of randomised intervention and control groups.^51^ Ten studies were quasi-experimental studies^31,33,52–59^ and nine were qualitative studies.^32,34,35,38,39,60–63^ Notably, eight of the quantitative studies also reported qualitative findings (Supplementary Table 3).

All included studies were conducted in Asia (*n* = 16) and Africa (*n* = 18). Among studies conducted in Asia, ten were from India, three were from China and one each was from Indonesia, Pakistan and Turkey. Among studies conducted in Africa, seven were from Zimbabwe, five were from Kenya, two were from South Africa and one each was from Tanzania, Botswana, Cameroon and Uganda. Interventions were predominantly delivered in community settings (*n* = 22), with seven conducted in schools and five in a clinic or hospital setting (Supplementary Table 3).

The included studies primarily involved adolescents and young people, with ages ranging from 10 to 30 years, and with most samples concentrated in the 15- to 24-year range. Participants were recruited from both rural and urban, school, community, university and clinic-based settings. Several studies focused on specific subpopulations, including adolescents and young people living with HIV, adolescent girls and young women, adolescent mothers and pregnant women, youth with psychological distress or diagnosed mental health conditions, and other vulnerable groups such as refugees, youth with substance use risk or chronic illness.

Most of the included studies reported group-based interventions (*n* = 24), peer-delivered or peer-facilitated counselling (*n* = 11) and psychoeducation (*n* = 8). Some studies combined elements such as group-based counselling^34–37,43,45,47^ or group-based self-help groups.^52,53^ Cognitive-based therapy (*n* = 9) and mindfulness-based training (*n* = 3) were among the approaches incorporated in peer-facilitated counselling interventions (Supplementary Table 3).

Youth peers performed roles as leaders (*n* = 6), facilitators (*n* = 10) or educators (*n* = 5) in many of the included studies. In 13 studies, youth peers were trained to deliver counselling or structured psychotherapy, facilitate group-based interventions, provide psychoeducation, support screening and brief interventions and offer community-based adherence or wellness support. In a few studies, peers also served as sources of informal support^53^ or assisted with participant recruitment (Supplementary Table 3).^52^ Across studies, peers were recruited using predetermined criteria that extended beyond age proximity and lived experience of mental health challenges, to include embeddedness, communication skills, leadership qualities and interest in supporting youth well-being. Additional characteristics included being students, community youth leaders or near-peer lay counsellors, as well as adolescents and young adults living with HIV, individuals with other chronic conditions, or former or active substance users.

### Quality appraisal

#### RCTs

Overall, the methodological quality of the RCTs was mixed (Table 1). Domain-level appraisal showed that randomisation and allocation procedures were variable, with 9 out of 15 trials achieving scores at or above 25 out of 30. Blinding of participants and outcome assessors was consistently weak across all trials. In contrast, outcome measurement and follow-up were generally strong, with 12 out of 15 trials receiving scores of 25 out of 25. Analytical rigour and reporting quality were also relatively robust, with 10 out of 15 trials scoring at or about 25 out of 30. Based on the overall ratings, eight RCTs were classified as high quality and the rest as moderate (*n* = 6) and low quality (*n* = 1).

**Table 1:** Critical appraisal of included randomised controlled trials (RCTs)

	JBI Items													Domain scores				Overall	
Study	1	2	3	4	5	6	7	8	9	10	11	12	13	A	B	C	D	Score	Quality
Balaji et al, 2011^40^	Y	U	Y	U	N	U	Y	Y	Y	Y	Y	N	N	20	3	25	17	65	Moderate
Beji-Chauke et al, 2025^37^	Y	N	Y	N	N	N	Y	Y	U	Y	Y	Y	Y	25	10	25	17	77	High
Bhatia et al, 2023^42^	Y	N	Y	N	N	Y	Y	Y	Y	Y	Y	Y	Y	25	13	25	25	88	High
Ding and Yao, 2020^44^	Y	U	Y	U	U	U	Y	Y	Y	Y	Y	Y	Y	20	0	25	25	70	Moderate
Dow et al, 2018^45^	Y	U	U	N	N	U	Y	Y	Y	Y	Y	Y	Y	10	7	25	25	67	Moderate
Fuhr et al, 2019^11^	Y	Y	Y	N	N	Y	Y	Y	Y	Y	Y	Y	Y	30	7	25	25	87	High
Gayathri et al, 2024^47^	Y	Y	U	N	N	U	Y	U	U	Y	Y	Y	N	25	3	22	13	63	Moderate
Keyan et al, 2025^48^	Y	Y	Y	N	N	Y	Y	Y	Y	Y	Y	Y	Y	30	7	25	25	87	High
Li et al, 2024^49^	Y	U	Y	N	N	U	Y	Y	Y	Y	Y	Y	Y	25	3	25	25	78	High
Maselko et al, 2020^41^	Y	Y	Y	U	N	Y	Y	Y	Y	Y	Y	Y	Y	30	10	25	25	90	High
Nasution et al, 2019^51^	Y	U	Y	U	U	U	Y	Y	Y	Y	Y	Y	Y	25	10	25	25	85	High
Ndongo et al, 2025^50^	Y	U	N	Y	U	Y	Y	N	N	Y	Y	N	Y	15	17	19	8	59	Moderate
Rodriguez et al, 2021^46^	Y	U	Y	U	U	U	Y	Y	Y	Y	Y	Y	Y	25	10	25	25	85	High
Simms et al, 2022^36^	Y	N	Y	N	N	U	Y	Y	Y	Y	Y	Y	Y	20	3	25	25	73	Moderate
Vancampfort et al, 2025^43^	U	U	N	N	N	Y	Y	U	N	Y	Y	Y	U	10	7	16	8	41	Low

Domains	Weighting, %	JBI items, Y (yes = 2), U (unclear = 1), N (no = 0)
A. Randomisation and allocation	30	Was true randomisation used for assignment of participants to treatment groups? Was allocation to treatment groups concealed? Were treatment groups similar at the baseline?
B. Blinding	20	Were participants blind to treatment assignment? Were those delivering treatment blind to treatment assignment? Were outcomes assessors blind to treatment assignment?
C. Outcome measurement and follow-up	25	Were treatment groups treated identically other than the intervention of interest? Was follow-up complete and if not, were differences between groups in terms of their follow up adequately described and analysed? Were outcomes measured in the same way for treatment groups? Were outcomes measured in a reliable way?
D. Analysis and reporting	25	Were participants analysed in the groups to which they were randomised? Was appropriate statistical analysis used? Was the trial design appropriate, and any deviations from the standard randomised controlled trial design (individual randomisation, parallel groups) accounted for in the conduct and analysis of the trial?

The methodological quality of each randomised controlled trial was assessed using the JBI Critical Appraisal Checklist for RCTs. Individual item scores (0 = N, 1 = U, 2 = Y) were grouped into four domains: randomisation and allocation, blinding, outcome measurement and follow-up, and analysis and reporting. For each domain, item scores were summed and converted to a percentage of the maximum possible domain score. Domain percentages were then multiplied by prespecified domain weights to generate weighted domain scores. The overall methodological quality score represents the sum of all weighted domain scores, expressed as a percentage. Studies were classified as high quality (≥75%), moderate quality (50–74%) or low quality (<50%). Consistent with JBI guidance, these scores reflect methodological quality rather than risk of bias. JBI, Joanna Briggs Institute; N, no; U, unclear; Y, yes.

#### Quasi-experimental studies

Overall, the methodological quality of the quasi-experimental studies was mixed (Table 2). Domain-level appraisal indicated uniformly strong performance in the cause-and-effect domain, with all ten studies achieving full scores (25/25). In contrast, participant comparability across study groups was limited, with only two out of ten studies receiving full scores in this domain. Outcome measurement was also a common area of weakness, as all studies scored below 20 out of 25. Statistical analysis showed mixed rigour, with only five out of ten studies achieving full scores. Overall, five studies were classified as high quality and five as moderate quality.

**Table 2:** Critical appraisal of included quasi-experimental or non-randomised studies of intervention

Study	1	2	3	4	5	6	7	8	9	A	B	C	D	Score	Quality
Chingono et al, 2022^52^	Y	Y	Y	N	N	Y	Y	Y	N	25	17	19	0	60	Moderate
Chory et al, 2022^53^	Y	Y	Y	N	N	Y	Y	Y	N	25	17	19	0	60	Moderate
Eryılmaz 2017^54^	Y	Y	Y	Y	N	Y	Y	Y	Y	25	25	19	25	94	High
Harrison et al, 2023^57^	Y	Y	Y	Y	N	N/A	Y	Y	Y	25	25	13	25	88	High
Im et al, 2018^55^	Y	Y	Y	N	N	Y	Y	Y	Y	25	17	19	25	85	High
Kavya et al, 2020^56^	Y	N	U	Y	N	U	Y	Y	N	25	13	16	0	53	Moderate
Kermode et al, 2021^33^	Y	Y	Y	N	N	Y	Y	Y	Y	25	17	19	25	85	High
Machisa et al, 2023^58^	Y	Y	U	N	N	N	N	Y	U	25	13	6	13	56	Moderate
Mathias 2018 et al,^31^	Y	Y	Y	N	N	Y	Y	Y	Y	25	17	19	25	85	High
Tinago et al, 2024^59^	Y	N	U	Y	N	Y	Y	Y	N	25	13	19	0	56	Moderate

Domains	Weighting, %	JBI items, Y (yes = 2), U (unclear = 1), N (no = 0)
A. Cause and effect	25	Is it clear in the study what is the ‘cause’ and what is the ‘effect’?
B. Participant comparability	25	Were the participants included in any comparisons similar? Were the participants included in any comparisons receiving similar treatment/care, other than the exposure or intervention of interest? Was there a control group?
C. Outcome measurement	25	Were there multiple measurements of the outcome both pre and post the intervention/exposure? Was follow-up complete and if not, were differences between groups in terms of their follow-up adequately described and analysed? Were the outcomes of participants included in any comparisons measured in the same way? Were outcomes measured in a reliable way?
D. Statistical analysis	25	Was appropriate statistical analysis used?

The methodological quality of each non-randomised study of intervention was assessed using the JBI Critical Appraisal Checklist for non-randomised studies of intervention. Individual item scores (0 = N, 1 = U, 2 = Y) were grouped into four domains: cause and effect, participant comparability, outcome measurement and statistical analysis. For each domain, item scores were summed and converted to a percentage of the maximum possible domain score. Domain percentages were then multiplied by prespecified domain weights to generate weighted domain scores. The overall methodological quality score represents the sum of all weighted domain scores, expressed as a percentage. Studies were classified as high quality (≥75%), moderate quality (50–74%) or low quality (<50%). Consistent with JBI guidance, these scores reflect methodological quality rather than risk of bias. JBI, Joanna Briggs Institute; N, no; U, unclear; Y, yes.

#### Qualitative studies

Overall, the methodological quality of the nine qualitative studies and eight quantitative studies with qualitative components was high (Table 3). Domain-level appraisal demonstrated strong congruity between the research questions, methodology and methods, with 16 out of 17 studies receiving full scores (25/25) in both the research congruity and methodology and methods domains. Interpretation and conclusions were likewise robust, with 16 out of 17 studies achieving full scores. In contrast, representation and ethical considerations were less consistently addressed, as only 7 out of 17 studies received full scores in this domain. Based on the proportion of JBI criteria met across domains, all but one study was classified as high quality (16 out of 17).

**Table 3:** Critical appraisal of qualitative studies and findings

Study	1	2	3	4	5	6	7	8	9	10	A	B	C	D	Score	Quality
Beji-Chauke et al, 2025^37^	U	Y	Y	Y	Y	U	U	Y	Y	Y	25	25	19	25	94	High
Chingono et al, 2022^52^	Y	Y	Y	Y	Y	U	N	Y	Y	Y	25	25	16	25	91	High
Chory et al, 2022^53^	Y	Y	Y	Y	Y	Y	U	Y	Y	Y	25	25	22	25	97	High
Dhand 2006^60^	Y	Y	Y	Y	Y	N	Y	U	N	Y	25	25	9	25	84	High
Dow et al, 2018^45^	U	Y	Y	Y	Y	U	U	U	Y	Y	25	25	16	25	91	High
Eryılmaz 2017^54^	Y	Y	Y	Y	Y	U	U	Y	U	Y	25	25	16	25	91	High
Ferris France et al, 2023^62^	Y	Y	Y	Y	Y	U	Y	Y	Y	Y	25	25	22	25	97	High
Garriot et al, 2023^63^	U	Y	Y	Y	Y	U	Y	Y	Y	Y	25	25	22	25	97	High
Harrison et al, 2023^57^	U	Y	Y	Y	Y	U	U	Y	Y	Y	25	25	19	25	94	High
Jaguga et al, 2023^38^	U	Y	Y	Y	Y	U	Y	Y	Y	Y	25	25	22	25	97	High
Jaguga et al, 2025^39^	U	Y	Y	Y	Y	U	Y	Y	Y	Y	25	25	22	25	97	High
Machisa et al, 2023^58^	U	Y	Y	Y	Y	U	Y	Y	Y	Y	25	25	22	25	97	High
Mathias et al, 2019^32^	Y	Y	Y	Y	Y	U	U	Y	Y	Y	25	25	19	25	94	High
Simms et al, 2022^36^	Y	Y	Y	Y	Y	U	U	Y	Y	Y	25	25	19	25	94	High
Wambua et al, 2019^61^	U	Y	Y	U	U	Y	Y	U	Y	U	13	13	22	13	59	Moderate
Wogrin et al, 2019^34^	U	Y	Y	Y	Y	U	Y	U	Y	Y	25	25	19	25	94	High
Wogrin et al, 2021^35^	U	Y	Y	Y	Y	U	Y	U	Y	Y	25	25	19	25	94	High

Domains	Weighting, %	JBI items, Y (yes = 2), U (unclear = 1), N (no = 0)
A. Research congruity	25	Is there congruity between the stated philosophical perspective and the research methodology? Is there congruity between the research methodology and the research question or objectives?
B. Methodology and methods	25	Is there congruity between the research methodology and the methods used to collect data? Is there congruity between the research methodology and the representation and analysis of data?
C. Representation and ethics	25	Is there a statement locating the researcher culturally or theoretically? Is the influence of the researcher on the research, and *vice versa*, addressed? Are participants, and their voices, adequately represented? Is the research ethical according to current criteria, and is there evidence of ethical approval?
D. Interpretation and conclusions	25	Is there congruity between the research methodology and the interpretation of results? Do the conclusions drawn in the research report flow from the analysis or interpretation of the data?

The methodological quality of each qualitative study was assessed using the JBI Critical Appraisal Checklist for Qualitative Research. Individual item scores (0 = N, 1 = U, 2 = Y) were grouped into four domains: research congruity, methodology and methods, representation and ethics, and interpretation and conclusions. For each domain, item scores were summed and converted to a percentage of the maximum possible domain score. Domain percentages were then multiplied by prespecified domain weights to generate weighted domain scores. The overall methodological quality score represents the sum of all weighted domain scores, expressed as a percentage. Studies were classified as high quality (≥75%), moderate quality (50–74%) or low quality (<50%). Consistent with JBI guidance, these scores reflect methodological quality rather than risk of bias. JBI, Joanna Briggs Institute; N, no; U, unclear; Y, yes.

### Intervention effects from quantitative studies

#### Counselling

Five studies evaluated peer-based counselling interventions, including two individual RCTs, two cluster RCTs and one controlled pre–post study. Across outcomes, depression and anxiety were most commonly assessed. The two high-quality RCTs reported consistently beneficial effects in the small to moderate range, although only one outcome, depression at 3 months, reached statistical significance.^11^ One cluster RCT, appraised as moderate quality, demonstrated moderate to large beneficial effects,^36^ whereas the other high-quality cluster RCT reported negligible to moderate effects.^37^ Overall, peer-based counselling interventions showed a consistent pattern of beneficial effects for depression and common mental disorder outcomes, with less consistent evidence for improvements in anxiety.

#### Peer support groups

Six studies examined peer-support groups, including two RCTs, two controlled pre–post studies and two uncontrolled pre–post designs. Findings from the RCTs generally trended toward beneficial effects, although only one trial reported a statistically significant, moderate-sized effect.^49^ Non-randomised studies similarly suggested beneficial trends, often with larger observed effects than those reported in RCTs, with the largest effects emerging from moderate-quality controlled^59^ and uncontrolled pre–post studies.^52^ No clear pattern of differential effectiveness by outcome type was observed, with effects varying across symptom domains.

#### Psychosocial interventions

Five studies assessed peer-led psychosocial interventions, comprising one RCT, two cluster RCTs and two uncontrolled pre–post studies. The RCT reported moderate beneficial effects that did not reach statistical significance, likely reflecting limited statistical power.^47^ Cluster RCTs showed effects trending toward benefit, ranging from negligible to moderate; negligible effects were observed in a high-quality trial,^41^ whereas moderate effects were reported in a large-sample cluster RCT^43^ appraised as lower quality. Overall, psychosocial interventions demonstrated a general pattern of beneficial effects for both anxiety and depression, although estimates varied by study design and methodological quality.

#### Psychoeducation

Four studies evaluated peer-led psychoeducation interventions, including two RCTs, one cluster RCT and one pre–post study. Beneficial effects for anxiety and depression were reported in one moderate-quality RCT,^44^ whereas the remaining studies found no notable effects. Evidence for the effectiveness of psychoeducation interventions seemed limited and less consistent than for other intervention types.

### Acceptability and feasibility of the intervention based on qualitative findings

Not all of the included studies addressed feasibility or acceptability, and those that did reported mixed findings (Supplementary Table 4). Some reported positive aspects like good treatment fidelity and adherence,^11,37,45,46^ and good acceptability of intervention formats that fostered safety and anonymity (e.g. WhatsApp).^53^ Participants described peer-led sessions as enjoyable because peers were perceived as easier to talk to, more relatable, and more understanding than professional healthcare workers.^37,38,57,63^ Participants also reported that the sessions were helpful in addressing and resolving challenges such as substance use and involvement in harmful relationships.^38,39,58^ Others highlighted challenges that could affect feasibility and acceptability. These challenges included stigma associated with using mental health services,^32,63^ limited access to devices, technology or the intervention itself, and insufficient programme resources to support participants.^33^ Complex family dynamics, such as parents withholding permission or making it conditional on completion of chores, further complicated participation.^32,33,36,39^ Lastly, competing demands on time,^33,39,61^ diverse educational backgrounds of participants^61^ and difficulty maintaining participant motivation over time^41,61,63^ were reported to have impacted acceptability and feasibility. These findings underscore the need to consider the specific context and challenges faced by the target youth population in the design and implementation of Y-PBMHPS.

### Factors that contribute to the success of Y-PBMHPS based on qualitative findings

A few studies in this review highlighted factors that contributed to the success of Y-PBMHPS. One key factor was the shared experiences among youth peers that fostered vulnerability and open communication,^34,37^ and that helped create a safe and supportive space for participants.^32,57^ Another factor was the positive connections and supportive community nurtured by these programmes, which helped reduce feelings of isolation and served as a resource for coping and building resilience.^45,57,61^ A third factor was the use of fun, easy and practical activities that equipped participants with knowledge and skills that boost self-agency, increased self-awareness, enhanced communication skills and induced a sense of accomplishment from helping others.^37,38,54,58,62^ Lastly, the programmes provided role-modelling opportunities where peers who demonstrated independence and competence become inspiration for others to learn and do the same.^32^

## Discussion

In this study, we reviewed the existing literature on peer-led or peer-facilitated programmes for youth mental health in LMICs. Our examination of both quantitative and qualitative data from the included studies indicates that Y-PBMHPS in educational and community settings can be feasible, acceptable and effective. The included studies are heterogeneous with respect to the nature of programmes and supports investigated, the roles assumed by youth peers, study settings and methodological quality. Nevertheless, a consistent finding across included studies was the reporting of marked reductions in psychological symptoms, notably depression and anxiety. Overall, the existing evidence underscores the potential of Y-PBMHPS as a promising avenue for broadening the scope of mental health support for youth in resource-constrained environments.

To date, and to our knowledge, only two review studies have focused on Y-PBMHPS with a scope similar to ours. The first, published in 2021,^12^ synthesised school-based mental health interventions for individuals between 4 and 18 years, based on studies available up to 12 May 2020. It identified a diverse array of study designs and peer roles; and, of the 54 included studies, only 11 assessed intervention efficacy, and just six were conducted in LMICs. The second, a scoping review published in 2025, examined peer-led mental health interventions in LMICs published as of September 2024.^64^ It emphasised the role of training, supervision and cultural competence in shaping programme implementation, but did not systematically assess the range of reported outcomes or intervention types. Although our review shares some thematic overlap with these prior syntheses, it makes a distinct and complementary contribution by systematically mapping the breadth of reported intervention effects and identifying key gaps in both the types of interventions evaluated and the methodological rigour of existing studies. This nuanced focus enhances the current evidence base and offers targeted directions for future research and practice in LMIC contexts.

The studies included in this review suggest that a variety of Y-PBMHPS are generally feasible and show trends toward beneficial effects, although the magnitude and consistency of impact vary by intervention type and study quality. Peer-based counselling demonstrated the most consistent benefits, particularly for depression and common mental disorder outcomes, whereas effects for anxiety were less consistent. Psychosocial and support group interventions also tended to show beneficial trends, although estimates varied widely across designs and were often smaller in high-quality randomised trials compared with non-randomised studies. In contrast, evidence for peer-led psychoeducation was limited and largely inconsistent, with beneficial effects observed in one moderate-quality trial. Overall, the pattern of smaller and frequently non-significant effects in high-quality RCTs, relative to larger effects reported in lower-quality or pre–post designs, underscores the need for more rigorous, adequately powered trials to draw definitive conclusions about effectiveness.

A feature of the Y-PBMHPS that this review underscores is the importance of adequate training and supervision for peers, as these will help achieve the interventions’ purported benefits and ensure the safety of peer facilitators and service recipients. Most of the studies included in this review described a form of training lasting from 8 h^46^ to 40 h^11^ that peers must undergo before they are deployed to deliver the intervention (Table 1). In one of the included studies,^32^ monthly refresher training was provided to peers over a 5-month intervention period, along with ongoing supervision from the project staff. Some Y-PBMHPS, such as peer support groups described in two studies,^53,61^ also involved the presence of mental health professionals, likely indicative of the need to protect the safe spaces provided by peer support groups from participants who could inadvertently threaten them. As others have noted, developing guidelines for selecting, training and supervising youth peers^12^ would be critical to the effective and safe delivery of Y-PBMHPS.

There is a notable scarcity of published studies from other LMICs, particularly in South American countries. It is likely that some form of evaluation and assessment are happening to monitor and assess Y-PBMHPS in these countries, and that programme proponents may either not submit their evaluations for peer review or publish them in languages other than English. This lack of accessible, peer-reviewed literature from other LMICs is an important gap that future studies should address to help build the evidence base for Y-PBMHPS in low-resource settings. Expanding the evidence base will also support the delivery of effective programmes with high implementation fidelity.

We note some limitations of this review. First, it was not possible for this rapid review to search for non-English publications, and this may have affected the diversity of the evidence included and assessed, such as those coming from South America. Additionally, the methodology excluded reports and documents that were not peer reviewed, omitting potentially valuable insights from the grey literature. Furthermore, the review criteria did not cover interventions or programmes that are active but remain unevaluated or those with evaluations that are ongoing and have yet to be published. This review, therefore, may have overlooked a segment of evidence, including those that are just emerging, that could offer important insights into the scope and impact of peer-led mental health initiatives. Incorporating environmental scans and informal searches of organisational reports and documents to capture a broader spectrum of evidence could help mitigate this limitation in the future.

In conclusion, the body of evidence synthesised in this review suggests that Y-PBMHPS represent a promising avenue for broadening the scope of mental health support in resource-constrained environments. To harness their full potential and ensure safety, it is imperative to establish comprehensive guidelines for selecting, training, supporting and supervising peer facilitators. The unique context and challenges of the target youth population must also be considered in their design and implementation. Further research in other LMICs and conducting health economic and cost-effectiveness studies could help broaden the evidence base and enhance the robustness of these programmes.

## Supporting information

10.1192/bjo.2026.11030.sm001 Puyat et al. supplementary material 1 Puyat et al. supplementary material

10.1192/bjo.2026.11030.sm002 Puyat et al. supplementary material 2 Puyat et al. supplementary material

10.1192/bjo.2026.11030.sm003 Puyat et al. supplementary material 3 Puyat et al. supplementary material

10.1192/bjo.2026.11030.sm004 Puyat et al. supplementary material 4 Puyat et al. supplementary material

## Data Availability

Data availability is not applicable to this article as no new data were created or analysed in this study.

## Supplementary material

The supplementary material is available online at https://doi.org/10.1192/bjo.2026.11030
