# Supplementary material for: Youth peer-based mental health programmes and supports in low- and middle-income countries: rapid review
Source: BJPsych Open. 2026 May 6;12(3):e126. doi: 10.1192/bjo.2026.11030 (PMC13150721; doi:10.1192/bjo.2026.11030)
Supplement: Puyat et al. supplementary material 1 — Puyat et al. supplementary material [file S2056472426110308sup001.docx]

**Supplementary Material. Full search strategy**

**1.1. Ovid MEDLINE (search run July 29, 2022)**

| **#** | **Search term** | **Results** |
| --- | --- | --- |
| 1 | mental disorders/ or exp anxiety disorders/ or exp "bipolar and related disorders"/ or exp mood disorders/ or exp substance-related disorders/ | 672,352 |
| 2 | Mental Health/ | 55,179 |
| 3 | ((mental* or psychological or psychiatr*) adj3 (health or litera* or outcome* or illness* or wellbeing or well-being or wellness)).mp. | 315,309 |
| 4 | 1 or 2 or 3 | 893,898 |
| 5 | (Peer* adj4 (support* or led or lead* or deliver* or run* or held or direct* or based or mentor* or assist* or facilitat*)).mp. | 17,374 |
| 6 | (program* or intervention* or workshop* or train* or psychoeducation).mp. | 2,753,418 |
| 7 | (adolescen* or youth* or young or emerging adult* or teen* or student*).mp. | 3,355,013 |
| 8 | 4 and 5 and 6 and 7 | 1,022 |
| 9 | limit 8 to (english language and yr="2002 -Current") | 934 |

Updated search run September 19, 2025

Limits:

(202207* or 202208* or 202209* or 20221* or 2023* or 2024* or 2025*).dt,ez,da.

608 new results (including 30 duplicates)

**1.2. Web of Science (search run July 29, 2022)**

**(Social Science Citation Index and Science Citation Index Expanded)**

| **#** | **Search term** | **Results** |
| --- | --- | --- |
| 1 | ALL=(mental health) | 778,716 |
| 2 | ALL=(mental disorders) | 338,363 |
| 3 | TS=((mental* or psychological or psychiatr*) NEAR/3 (health or litera* or outcome* or illness* or wellbeing or well-being or wellness)) | 238,069 |
| 4 | #3 OR #2 OR #1 | 901,621 |
| 5 | TS=(peer* NEAR/4 (support* or led or lead* or deliver* or run* or held or direct* or based or mentor* or assist* or facilitat*)) | 33,727 |
| 6 | ALL=(program* or intervention* or workshop* or train* or psychoeducation) | 10,487,962 |
| 7 | ALL=(adolescen* or youth* or young or emerging adult* or teen* or student*) | 4,049,999 |
| 8 | #4 AND #5 AND #6 AND #7 Timespan: 2002-01-01 to 2022-07-29 | 1,241 |

Updated search run September 19, 2025

Limits:

LD=(2022-07-30/2025-09-19)

**Article** or **Review Article** (Document Types) and **English** (Languages)

842 new results (including 346 duplicates)

**1.3. PsycINFO (EBSCO) (search run July 29, 2022)**

| **#** | **Search term** | **Results** |
| --- | --- | --- |
| 1 | (mental health) OR (mental disorders) OR ((mental* or psychological or psychiatr*) N3 (health or litera* or outcome* or illness* or wellbeing or well-being or wellness)) | 890,008 |
| 2 | peer* N4 (support* or led or lead* or deliver* or run* or held or direct* or based or mentor* or assist* or facilitat*) | 21,796 |
| 3 | program* or intervention* or workshop* or train* or psychoeducation | 1,320,570 |
| 4 | adolescen* or youth* or young or emerging adult* or teen* or student* | 1,440828 |
| 5 | 1 AND 2 AND 3 AND 4 | 1,655 |
| 6 | 1 AND 2 AND 3 AND 4  Language : English  Limiters : Publication date: 20020101 - 20220731 | 1,505 |

Updated search run September 19, 2025

Limits:

English, Academic Journals, Publication Year: 2022-2026

505 new results (including 331 duplicates)

**1.4. CINAHL (search run July 29, 2022)**

| **#** | **Search term** | **Results** |
| --- | --- | --- |
| 1 | (mental health) OR (mental disorders) OR ((mental* or psychological or psychiatr*) N3 (health or litera* or outcome* or illness* or wellbeing or well-being or wellness)) | 276,541 |
| 2 | peer* N4 (support* or led or lead* or deliver* or run* or held or direct* or based or mentor* or assist* or facilitat*) | 13,740 |
| 3 | program* or intervention* or workshop* or train* or psychoeducation | 1,234,024 |
| 4 | adolescen* or youth* or young or emerging adult* or teen* or student* | 1,096,032 |
| 5 | 1 AND 2 AND 3 AND 4 | 524 |
| 6 | 1 AND 2 AND 3 AND 4  Language : English  Limiters : Publication date: 20020101 - 20220731 | 503 |

Updated search run September 19, 2025

Limits:

(EM 20220730- OR (ZD "in process" AND RD 20220730-))

338 new results (including 224 duplicates)

**1.5. CAB Direct (search run July 29, 2022)**

| **#** | **Search term** | **Results** |
| --- | --- | --- |
| 1 | [(mental health) OR (mental disorders) OR ((mental* or psychological or psychiatr*) 3 (health or litera* or outcome* or illness* or wellbeing or well-being or wellness)) AND yr:[2002 TO 2022]](https://www.cabdirect.org/cabdirect/search/?q=(mental%20health)%20OR%20(mental%20disorders)%20OR%20((mental*%20or%20psychological%20or%20psychiatr*)%203%20(health%20or%20litera*%20or%20outcome*%20or%20illness*%20or%20wellbeing%20or%20well-being%20or%20wellness))%20AND%20yr:%5B2002%20TO%202022%5D&sort=Relevance) | 113,149 |
| 2 | peer* 4 (support* or led or lead* or deliver* or run* or held or direct* or based or mentor* or assist* or facilitat*) | 9,047 |
| 3 | (program* or intervention* or workshop* or train* or psychoeducation) | 1,229,810 |
| 4 | adolescen* or youth* or young or emerging adult* or teen* or student* | 207,297 |
| 5 | (adolescen* or youth* or young or emerging adult* or teen* or student*) AND ((program* or intervention* or workshop* or train* or psychoeducation)) AND (peer* 4 (support* or led or lead* or deliver* or run* or held or direct* or based or mentor* or assist* or facilitat*)) AND ((mental health) OR (mental disorders) OR ((mental* or psychological or psychiatr*) 3 (health or litera* or outcome* or illness* or wellbeing or well-being or wellness)) AND yr:[2002 TO 2022]) | 203 |

CAB Direct no longer exists, so the search update was run in the new interface, CABI Digital Library, on September 19, 2025. Terms were adapted for the new interface:

(adolescen* OR youth* OR young or "emerging adult" OR "emerging adults" OR teen* OR student*) AND (program* OR intervention* OR workshop* OR train* OR psychoeducation) AND ("peer support"~4 OR "peer supports"~4 OR "supportive peer"~4 OR "peer led"~4 OR "peer leader"~4 OR "peer leaders"~4 OR "peer delivery"~4 OR "peer delivered"~4 OR "peer run"~4 OR "peer held"~4 OR "peer directed"~4 OR "peer based"~4 OR "peer mentor"~4 OR "peer mentors"~4 OR "peer mentorship"~4 OR "peer assisted"~4 OR "peer assistance"~4 OR "peer facilitated"~4 OR "peer facilitator"~4 OR "peer facilitators" OR "peers support"~4 OR "peers supports"~4 OR "supportive peers"~4 OR "peers led"~4 OR "peers leader"~4 OR "peers leaders"~4 OR "peers delivery"~4 OR "peers delivered"~4 OR "peers run"~4 OR "peers held"~4 OR "peers directed"~4 OR "peers based"~4 OR "peers mentor"~4 OR "peers mentors"~4 OR "peers mentorship"~4 OR "peers assisted"~4 OR "peers assistance"~4 OR "peers facilitated"~4 OR "peers facilitator"~4 OR "peers facilitators") AND ("mental disorders" OR "mental health"~3 OR "mental literacy"~3 OR "mental outcomes"~3 OR "mental illness"~3 OR "mental illnesses"~3 OR "mental wellbeing"~3 OR "mental well-being"~3 OR "mental wellness"~3 OR "psychological disorders" OR "psychological health"~3 OR "psychological literacy"~3 OR "psychological outcomes"~3 OR "psychological illness"~3 OR "psychological illnesses"~3 OR "psychological wellbeing"~3 OR "psychological well-being"~3 OR "psychological wellness"~3 OR "psychiatric disorders" OR "psychiatric health"~3 OR "psychiatric literacy"~3 OR "psychiatric outcomes"~3 OR "psychiatric illness"~3 OR "psychiatric illnesses"~3 OR "psychiatric wellbeing"~3 OR "psychiatric well-being"~3 OR "psychiatric wellness"~3)

Limited to Global Health database, English, publication years 2022-2025

73 new results (including 50 duplicates)
