# Supplementary material for: Youth peer-based mental health programmes and supports in low- and middle-income countries: rapid review
Source: BJPsych Open. 2026 May 6;12(3):e126. doi: 10.1192/bjo.2026.11030 (PMC13150721; doi:10.1192/bjo.2026.11030)
Supplement: Puyat et al. supplementary material 3 — Puyat et al. supplementary material [file S2056472426110308sup003.docx]

**Table S2. Eligibility criteria based on the PICO framework**

| **PICO(s)** | **Included** | **Excluded** |
| --- | --- | --- |
| **Population** | Youth aged 15-30 years in low- or middle-income countries as classified by the World Bank | Individuals <15 years old or >30 years old  Not in LMIC country setting |
| **Intervention** | School, community or healthcare setting based, peer*-based mental health supports, programs or services  Any mode of delivery (in-person, online)  **Definition of a peer: Persons belonging to the same age group or those with lived experience of mental health challenges or illness. A peer may have additional specific characteristics (e.g., health status, bereavement, or other lived contexts) beyond age shared or experience of mental illness that may also shape the nature and mechanisms of support.* | Pharmacological interventions  Interventions that are not peer-based  Interventions that are exclusively provided by mental health professionals |
| **Comparator** | Any or none |  |
| **Outcomes** | **Quantitative:** Incidence, prevalence, or severity of mental illness*; Psychosocial outcomes** (e.g., quality of life, mental well-being, social functioning); Mental health-related knowledge, attitudes, health-seeking behaviour  **Definition of mental illness or mental health conditions: clinical outcomes classified in ICD-10 or DSM-IV, measured by a healthcare professional or using a validated tool (see Table S4)*  ***Definition of psychosocial outcomes: Changes to the psychological and social well-being of an individual (see Table S4)*  **Qualitative:** Experiences with the intervention; Perspectives about the intervention’s importance, feasibility, acceptability, accessibility |  |
| **Study Designs** | Randomized or non-randomized interventional studies  Qualitative studies on an intervention | Literature reviews  Observational studies/non-interventional studies |
| **Publication Type** | English  Published after 2002  Peer reviewed articles | Not English  Published before 2002  Book/book chapters, letters to the editor, commentaries, theses, dissertations |
