# Supplementary material for: Youth peer-based mental health programmes and supports in low- and middle-income countries: rapid review
Source: BJPsych Open. 2026 May 6;12(3):e126. doi: 10.1192/bjo.2026.11030 (PMC13150721; doi:10.1192/bjo.2026.11030)
Supplement: Puyat et al. supplementary material 4 — Puyat et al. supplementary material [file S2056472426110308sup004.docx]

# **Table S3. Summary of study characteristics**

| **Author and Year of Publication**  **Overall Quality Appraisal Score and Category** | **Country** | **Approach and methods** | **Number of Participants and Peers**  **and Participant Characteristics** | **Key Role of Peers**  **and Peer Definition** | **Type of Intervention** | **Intervention Summary** | **Training and Supervision for Peers** |
| --- | --- | --- | --- | --- | --- | --- | --- |
| Balaji 2011  65; Moderate | India | **Quantitative**  Cluster randomized trial | Rural:  Baseline(n= 1803)  Follow-up(n= 1620)  Urban:  Baseline(n=1860)  Follow-up(n= 1942)   Peer leaders  Community-based:  Rural (n = 28), Urban (n = 0)  School-based:  n= 98  Youth aged 16-24 (mean age 19) living in rural and urban communities. | Leaders  Peers were youth who were recruited based on a pre-determined criteria. Criteria not reported. | **Community/School-based;**  **Psychoeducation**  Yuva Mitr (friend of youth): a multicomponent (peer/teacher) health education intervention | 1) Peer education program involved training a selected number of youth (“peer leaders”) to provide health information to other youth in their communities.  2) In urban communities, the intervention also involved a teacher training program to improve teacher-student relationships, early detection and management of most common youth problems in school settings, and counseling skills.  3) Health information materials were developed by the research team and distributed to youth through house-to-house visits and posters.  4) A social worker, two psychologists, and three peer educators formed the intervention team. The intervention lasted 12 months, with an intensive engagement period in the first six months. | Peer leaders were trained by psychologists and social workers with expertise in adolescent health and were expected to conduct group sessions and perform street plays. This component was supported by village key council leaders in rural areas and by trained teachers in urban educational institutions. |
| Beji-Chauke 2025  Quantitative: 77; High  Qualitative:  94; High | Zimbabwe | **Quantitative + Qualitative**  Cluster randomized trial + qualitative component (interviews) | Interview (n=13)  Intervention group (n=187)  Control group (n=86)  Youth aged 16-19 who scored 8+ on SSQ-14. | Counsellor  Peers were undergraduate students of mixed sexes studying psychology and serving a 10-month attachment with the Friendship Bench. | **Community-based;**  **Counselling**  YouFB: Youth Friendship Bench group counselling led by peers | 1) 4-6 weekly sessions of problem-solving therapy based on Indigenous concepts: kuvhura, pfungwa, kusimudzira and kusimbisa  2) Included helping clients become aware of the problems they were experiencing. With support from YouFB buddies or an adult CHW, they then selected one issue to focus on, brainstormed possible solutions, chose one, and were ultimately empowered to create a detailed plan for putting the solution into action. | Peers were supervised by FB trainers, adult community health workers who were not delivering the intervention in this trial and clinicians. |
| Bhatia 2023  88; High | India | **Quantitative**  Cluster randomized trial | Intervention group (n=737)  Control group (n=741)  Peer educators (n=30)  Adolescent girls aged 10-19. | Educators  Female and male peer facilitators called *yuva saathi* (“friend of youth”), aged 20-25 years old. | **Community-based;**  **Psychoeducation**  Peer education on nutrition, health, mental health and violence | 1) Three-component intervention delivered by “Yuva saathi” young adult peer facilitators, adult youth leadership facilitators and adult livelihood promoters  2) Yuva Saathi facilitated monthly group meetings, using PLA (Participatory Learning & Action) cycles focused on education, nutrition, health and mental health, and violence.  3) Youth leadership activities were organized every 2 months to deliver activities boosting self-efficacy, resilience and social connectedness  4) Livelihood promoters met with adolescents every three months to provide adolescents with practical skills to improve their families’ food security and income. | The community youth team received periodic residential training in delivering the intervention using participatory approaches, communication skills, and working with adolescents and families. Training was supplemented with regular supervision. |
| Chingono 2022  Quantitative: 60; Moderate  Qualitative:  91; High | Zimbabwe | **Quantitative + Qualitative**  Pre-post without control + qualitative component (interviews and FGDs) | Interview (n=18)  Baseline survey (n= 93)  Follow-up survey (n=51)  Adolescent girls and young women aged 16-19 who reported selling sex as their main means of earning of livelihood and were either currently pregnant or had at least one child. | Educators/recruiter  Peers were active Sisters program educators, characteristics not reported | **Community-based;**  **Support group**  Self-help, peer education and practice groups | 1) A package of 12 participatory sessions delivered over 6 months was developed, including a facilitator’s manual and participant handbook to guide the sessions.  2) A trained facilitator coordinated all the sessions  3) Activities aimed to developing individual and group problem-solving skills, practice-based learning for healthy parenting and safer sexual practices, the importance of self-care and appropriate health-seeking, and improving capacity to manage and save money.  4) Seven SHGs were established. Each had 10–15 participants recruited by Sisters peer educators | N/A |
| Chory 2022  Quantitative:  60; Moderate  Qualitative:  97; High | Kenya | **Quantitative + Qualitative**  Pre-post without control + qualitative component (pre-post interviews) | Baseline (n=30) Follow-up (n=29)  Pre-intervention interview  (n=25)  Post-intervention interview  (n=15)  Adolescents living with HIV aged 10-19, who were on ART, engaged in HIV care and aware of their HIV status. | Informal Support  Peers were adolescents living with HIV, aged 10-19 years who were on ART, engaged in HIV care, and aware of their HIV status. They were placed in WhatsApp groups with other peers. | **Community-based;**  **Support group**  Peer group discussions on social media (WhatsApp) monitored by a counsellor | 1) Participants were assigned to one of two WhatsApp® groups, one for 9–14-year-olds and another for 15–19-year-olds, with 15 people in each group, based on their age.  2) A professional counsellor led the WhatsApp® groups in accordance with a predetermined curriculum in order to promote positive support among group members, introduce weekly discussion themes, and respond to questions from participants.  3) Participants were encouraged to use the WhatsApp® groups to chat casually with other group members while the study counsellor monitored these conversations. | The counselor reached out to each participant through direct WhatsApp® messages every other week during the study period to offer regular check-ins |
| Dhand 2006  84; High | India | **Qualitative**  Ethnography (participant observation and semi-structured interviews) | Peer educators (n=8)  Participants are the peers. | Educators  Peers were active or ex-users whose drug use careers were based in Yamuna Bazaar, aged 26 to 50, and had at least 2 years of work experience. | **Community-based;**  **Psychoeducation**  Peer education outreach program targeting heroin addicts | 1) The program employed current or former addicts from the community; peer educators were defined as ‘those who give knowledge in the field.  2) Clients were the homeless heroin users who commonly accessed the health and harm reduction programs provided by SHARAN. They were mainly living in the Yamuna Bazaar area and were not receiving treatment. The majority were male, with an average age of 30 (with a range of 15 to 60 years old).  3) Peer educators approached heroin addicts to discuss high-risk behaviours and diseases, encouraging visits to the SHARAN Centre for services. | Every two weeks, peer educators attended sessions led by an NGO leader who taught lessons on topics such as HIV transmission, HIV testing methods, and public perceptions of HIV. |
| Ding 2020  70; Moderate | China | **Quantitative**  RCT | Intervention group (n=70)  Control group (n=71)  Middle school students aged 12-18. | Educators  Peers were middle school student participants who demonstrated interest in serving as peer leaders. | **School-based;**  **Psychoeducation**  Peer education on health knowledge and behaviours concerning COVID-19 AND Physical exercise program (Aerobics) | 1) Peer-leaders were also asked to organize weekly peer-education seminars (online live webcasts) to promote peer education among classmates and friends.  2) The intervention program includes 8 weeks of aerobic activity scheduled three days a week. | Students who demonstrated a strong interest in serving as peer leaders underwent training and were instructed to instruct their peers by speaking with them. |
| Dow 2018  Quantitative:  67; Moderate  Qualitative: 91; High | Tanzania | **Quantitative + Qualitative**  RCT + qualitative feasibility study (session notes and interviews) | Intervention participants (n=58)  Youth living with HIV aged 12-24, attending adolescent HIV clinic and receiving ART, and who were aware of their HIV status. | Leaders  Peers were group leaders aged between 24-30 years, chosen based on mixed experiences. Leaders had lived HIV experience or prior experience in delivering MH intervention for children. They were fluent in Kiswahili and proficient in English. | **Community-based; Counselling**  Group counselling led by peers | 1) Sauti ya Vijana (SYV) consisted of 10 group sessions and two individual sessions. The first four group sessions encouraged youth to identify their stress and worries about living with HIV. They were taught to use CBT methods to identify and change negative thoughts, feelings, and behaviours and relaxation methods such as deep breathing and mindfulness practices to cope with distress.  2) Group sessions five to seven emphasized nurturing strong familial and social relationships.  3) Group sessions eight through ten incorporated prior session teachings to cultivate a safe and healthy living environment through choices influenced by stigma, education about HIV, disclosure, and values.  4) Group sessions were delivered in the native language, Kiswahili, and each lasted approximately 90 minutes. | Peer leaders completed an intensive two-week, in-country SYV training led by the principal investigator and a U.S.-based clinical psychologist, following a detailed script for each group meeting. They also practiced for each session under supervision. |
| Eryılmaz 2017  Quantitative:  94; High  Qualitative:  91; High | Turkey | **Quantitative + Qualitative**  Pre-post with control group + qualitative component (follow-up interviews) | Peer helpers (n=30)  Total (n= 60):  Experiment group (n=30),  Control group (n=30)  University students aged 18-23. | Counsellor  Peers were mixed-sex counsellor candidates who were studying psychological counseling and guidance. Their mean age was 21.85 years. | **School-based; Counselling**  Peer-helping counselling program | 1) The intervention consisted of 15 activities; 8 one-on-one interviews, and 7 homework assignments.  2) The activities were based on positive psychology, including: Mind control, Placing a safe distance, Three things that made me happy today, Five things that made me happy in the past, My future goals, Gratitude, Thankfulness, The reasons for my happiness, Positive perceptions of myself, People who support me in my life, A way of thinking positively, Pleasures of the past, Happiness-oriented behaviours, My flow list, I'm solving my problems | The peer helpers received 3 hours of education each week for 2 months (totaling 24 hours) on improving subjective well-being and conducting activities. |
| Ferris France 2023  97; High | Zimbabwe | **Qualitative**  Qualitative evaluation study (individual interviews and focus group discussions) | Intervention participants (n=62)  Peer coaches (n=15)  Adolescents and young people living with HIV aged 18-24. | Leaders  Peer Community Adolescent Treatment Supporters (CATS) were 18-24 years olds living with HIV, trained to deliver structured support groups, counselling and tailored community-based adherence support. | **Community-based;**  **Support group**  Peer-delivered inquiry-based stress reduction (IBSR) support groups | 1) 16 x 3 hour groups sessions including individual and pair work to improve management of specific emotional and psychological symptoms.  2) Each session contained a mix of theory, meditation, group and individual experiences of IBSR, music reflection and sharing of insights.  3) Intervention was also supported by a 156-page activity journal containing worksheets, exercises and homework. | Peer coaches were trained by local and international trained adult coaches, at a residential face-to-face, six-day immersive Training of Trainers.  Intervention sessions were supported by adult coaches. |
| Fuhr 2019  87; High | India | **Quantitative**  RCT | Intervention participants:  Baseline (n=140), 3-month follow-up (n=123), 6-month (n=122)  Control participants:  Baseline (n=140), 3-month follow-up (n=122), 6-month (n=129)  Women aged 18 years or older (mean age 25), in their second or third trimester of pregnancy. | Counsellor  Peers were laywomen (without mental health training) who had interest in helping other women in their community. They were middle aged with children, had similar sociodemographic background as participants, and had good communication skills. | **Community-based;**  **Counselling**  Peer-delivered behavioural activation sessions (Thinking Healthy Programme Peer-delivered (THPP)) | 1) THP's theoretical foundation is cognitive behavioural therapy (CBT), and it incorporates tactics such as behavioural activation, active listening, family involvement, guided discovery, and homework.  2) 6–14 individual sessions were delivered over 7–12 months in four phases, including prenatal phase, early infancy, middle infancy, and late infancy  3) Treatment completion was defined as receiving a minimum of six sessions, with at least one session in each of the four phases  4) The THPP peers were referred to as Sakhis and were middle-aged women with children who shared the same socioeconomic background as the participants and possessed strong communication skills | Peers received 25-40 hours of training classes followed by 2-month of internship |
| Garriott 2023  97; High | Botswana | **Qualitative**  Qualitative evaluation study (semi-structured interviews) | Intervention participants (n=22)  Peer counsellors (n=18)  Youth living with HIV aged 13-25, speaking English or Setswana and a patient at the clinic. | Counsellor  Near peer youth lay counsellors (NPYLC) were close in age (18-35 years) to clients and recruited by community leaders who worked with youth in other community capacities. | **Community-based;**  **Counselling**  Problem-solving therapy (PST) peer counselling intervention | 4-6 sessions of PST were delivered, varying based on how long it took to complete the seven steps of PST: clarifying and defining the problem; establishing an achievable goal; generating multiple solution alternatives; implementing decision making guidelines; choosing the preferred solution(s); implementing the preferred solution(s); and evaluation of the outcome | Lay counsellors received training in psychoeducation, PST, as well as in mental health first aid. |
| Gayathri 2024  63; Moderate | India | **Quantitative**  RCT | Intervention group(n=30)  Control group (n=30)  Adolescents aged 12-18 with a clinical diagnosis of social anxiety disorder. | Facilitator  Peer definition not reported. | **Hospital-based;**  **Psychosocial**  Peer-assisted therapy | 3 months focused on structured peer interactions, role-playing, group discussions and collaborative tasks designed to foster social skill development, reduce social isolation, and provide opportunities for experiential learning | Not reported |
| Harrison 2023  Quantitative:  88; High  Qualitative:  94; High | South Africa | **Quantitative + Qualitative**  Post-intervention with control + qualitative component (in-depth interviews) | Intervention group (n=20)  Control group (n=38)  Post-intervention interview (n=20)  Young people living with chronic illnesses aged 13-24 who were receiving care at the hospital. | Facilitator/Mentor  Peer mentors were volunteers who are also living with a chronic condition. | **Hospital-based; Support group**  Peer-led group support session | 1) Weekly peer-facilitated group session to build social networks that enhance psychosocial support; develop a sense of belonging with peers; create space where adolescents can share their experience(s) living with chronic illness; and build empathy among ALHIV and other conditions.  2) Groups are divided into structured topics related to general adolescent health and development and less structured social groups. | Sessions are overseen by clinicians from the hospital’s Adolescent Clinicians Group.  A peer mentor supervisor (psychologist or social worker) is present to observe all groups and provide support where necessary. |
| Im 2018  85; High | Kenya | **Quantitative**  Pre-post without control | Trained peer leaders (n=25)  Peer leaders who participated in the intervention (n=10)  Intervention participants (n=145)  Survey participants (n=141)  Moderate to severe PTSD (n=45)  None to low PTSD (n=96)  Somali youth refugees (mean age 20). | Leaders  Peers were community youth leaders in the Somali community. | **Community-based;**  **Psychoeducation**  Peer-led Trauma-Informed Psychoeducation (TIPE) | 1) Peer-led intervention was provided in 12 sessions over three months and peer leaders were supervised by community health counsellors  2) TIPE training included education on the various impacts of trauma on the body, mind, social relationships, and spirituality, as well as psychosocial capacity such as emotional coping and problem solving, community and support systems, and conflict resolution skills | Peer leaders completed a week-long TIPE training of trainers (TOT) workshops |
| Jaguga 2023  97; High | Kenya | **Qualitative**  Qualitative evaluation study (focus group discussions and semi-structured interviews) | Intervention participants (n=100)  Focus group participants (n=25)  Peer providers (n=4)  Youth aged 15-24 enrolled at the clinic. | Counsellor  Peer providers work full-time at the clinic, and were selected based on age (18-24 years), HIV status (HIV positive, virally suppressed and ready to disclose status) and willingness to support youth wellness. | **Clinic-based; Counselling**  Peer-provided Screening and Brief Intervention program | 1) Screening using ASSIST-y (Alcohol Smoking and Substance Involvement Screening Test- Youth) questionnaire.  2) Brief intervention including either 5-10 minute session of positive reinforcement delivered to youth with no history of substance use over the past 3 months, or 20-30 minute brief motivational interviewing session for youth with moderate and high-risk substance use.  3) Youth with high-risk use received a referral to specialist care. | 5-day training (~30h) in HIV adherence counselling and basing counselling techniques, facilitated by pediatrician, clinical officers, psychologists and nursing staff stationed at the clinic. |
| Jaguga 2025  97; High | Kenya | **Qualitative**  Qualitative pilot study (individual semi-structured interviews) | Intervention participants (n=38)  Interview participants (n=25)  Peer provider (n=1)  Youth aged 15-24 with moderate-risk substance use. | Counsellor  The female peer provider (age 24) selected for this pilot RCT was trained to deliver brief intervention. | **Clinic-based; Counselling**  Peer-provided Screening and Brief Intervention | Single face-to-face 20-minute session delivered by a peer provider based on the FRAMES model and MI techniques, aimed to motivate intervention recipients to make changes regarding their substance use. | 5-day training (~30h) in HIV adherence counselling and basing counselling techniques, facilitated by pediatrician, clinical officers, psychologists and nursing staff stationed at the clinic. |
| Kavya 2020  53; High | India | **Quantitative**  Post- intervention with control (semi-structured questionnaire) | Bihar (n=345):  Participants (n= 180), Non-participants (n= 165) Jharkhand (n = 406):  Participants (n= 315), Non-participants (n=91)  Karnataka (n=267):  Participants (n=186), Non-participants (n=81)  Maharashtra (n=308):  Participants (n=287), Non-participants (n=21)  Adolescents aged 10-19. | Educators  Peer definition not reported. | **Community-based; Psychoeducation**  Peer-led intervention on knowledge, attitude and practices (KAP) related to sexual reproductive and mental health issues | 1) RMNCH+A (reproductive, maternal, newborn, child, and adolescent) service delivery platform was used to in this peer-led programs to increase adolescents' knowledge, attitude, practice, and behaviour around sexual reproductive health, nutrition, and common mental health disorders.  2) the project aimed to encourage behaviour change in young mothers and adolescents. It also created a community-wide framework to address continuity in information, counselling, and health-seeking behaviours. | N/A |
| Kermode 2021  85; High | India | **Quantitative**  Pre-post without control | Peer facilitator (n=8)  Intervention participants (n=142)  Young people recruited from the Burans project or through community word-of-mouth (mean age 18.9) | Facilitator  Peer facilitators were those affected by mental illness. They were recruited from local communities and selected based on their education level (at least Class 12), ability to travel to the intervention sites, and communication skills. | **Community-based; Support group**  Peer-facilitated, participatory intervention for young people affected by mental illness | 1) The intervention included 11 Youth Wellness Groups that were supported, community-based, peer-facilitated, and participative. 2) Over the course of 4-6 months, 9 female and 2 male groups (13 each) met 16 times, supervised by a series of interactive modules modified from the Nae Disha building youth resilience manual 3) The groups were facilitated by peers who had experienced mental illness and were actively supported by the project team. Two peer facilitators were recruited from local communities for each group, and they were chosen based on their level of education (at least Class 12), ability to travel to the intervention sites, and communication skills. | Peer facilitators received training and support by the Burans Team. |
| Keyan 2025  87; High | India | **Quantitative**  RCT | Coping with COVID (n=91)  Supportive Counselling (n=92)  University students with sufficient English comprehension, who scored 20+ on Kessler Psychological Distress Scale, without current psychosis or substance dependence. | Facilitator  Peer facilitators were recruited from school campuses across India, were completing their undergraduate studies in a discipline unrelated to psychology and had no prior experience in psychosocial programmes. | **School-based; Psychoeducation**  Peer-led psychoeducation program | 1) Coping with COVID consisted of 6 weekly group sessions of 60-minute duration, where the peer-facilitator guided participants on psychoeducation related to common reactions to COVID-19, stress management, and skills to strengthen social support.  2) non-directive Supportive Counselling consisted of 6 weekly group sessions of 60-minute duration, where the peer-facilitator led group discussions about how students were coping during the pandemic, ventilation of reactions to common problems experienced and possible solutions. | Peer facilitators received 8 days of training in basic counselling skills, delivery of Coping with COVID and supportive counselling techniques, group facilitation and self-care, by two clinical psychologists.  Weekly supervision was provided remotely by video teleconferencing. |
| Li 2024  78; High | China | **Quantitative**  RCT | Intervention group (n=45)  Control group (n=45)  Peer supporters (n=5)  Adolescent and young adult cancer patients (mean age 30). | Leader  Peer supporters were cancer patients of the same cohort as the patients, and met the following requirements: high school education or more, had surgery or chemoradiotherapy at least once, able to adhere to regular outpatient follow-up, and time to voluntarily help peer patients. | **Hospital-based; Support group**  Peer-facilitated group discussion sessions | 1) Intervention group participants received a manual on the intervention.  2) An 8-week online or offline intervention was conducted once a week, where peer supporters shared their experiences and assist patients in exploring solutions to their problems through their examples and practical experiences.  3) An online peer support WeChat group was established for information support. | Peers were trained through lectures, group discussions, and role-playing in 3 1-hr sessions |
| Machisa 2023  Quantitative:  56; Moderate  Qualitative:  97; High | South Africa | **Quantitative + Qualitative** pre-post without control + qualitative component (semi-structured interviews) | Intervention participants (n=98)  Interview participants (n=35)  First-year female students aged 18-30. | Facilitators  Peers were young women aged 18-30 years, a student or student support staff member in the selected institution, had passion and interest to learn about sexual violence and how to prevent it, and had confirmed availability. | **School-based; Psychosocial**  Peer-facilitated group discussion sessions | 1) NV! Is a sexual violence risk reduction group intervention of 10 weekly sessions of 3.5 hours each, delivered by 2 co-facilitators per group.  2) The session included participatory group and critical reflection methods to challenge gender inequitable beliefs, enhance personal life skills and stimulate reflection on strategies for sexual violence risk reduction. | Peer facilitators were trained by the research team in two sessions over 9 days and 7 days respectively. |
| Maselko 2020  90; High | Pakistan | **Quantitative**  Cluster randomized trial | Intervention participants:  Baseline (n=283)  36-months follow-up (n=206)  Control participants:  Baseline (n=287)  36-months follow-up (n=216)  Pregnant women in the third trimester, aged 18+ (mean age 26.7) and registered with their village Lady Health Workers. | Leaders  Peers were lay married women who lived in the same community as that of the depressed women and volunteered their time. | **Community-based; Psychosocial**  Peer-delivered psychosocial intervention (THPP+) including peer-support, behavioural activation, and problem-solving | 1) The intervention included 14 sessions (Phase 1: THPP, individual and group sessions) from pregnancy through six months after birth and 18 sessions (Phase 2: THPP+, booster group sessions) from 7 to 36 months after birth. The first six of the 18 "booster" sessions were provided monthly, followed by bimonthly sessions for the next 36 months.  2) Group meetings offered women a safe place to discuss their issues, exchange parenting advice, and provide one another with support.  3) Psychosocial intervention comprised non-specialist/peer support, behavioural activation, problem-solving in a culturally appropriate, nonmedicalized form, and developmental activities for children up to the 36th month | Peers received regular refresher training and supervision. |
| Mathias 2018  85; High | India | **Quantitative**  Pre-post without control | Intervention participants (n=106)  Young women aged 12-24 not attending school. | Facilitator  Peers were locally recruited young women aged 20-30 years who had interest in working in youth resilience and had completed 12^th^ class education. | **Community-based; Psychosocial**  Peer-facilitated weekly group sessions to promote youth development and resilience and reduce distress | 1) Nae Disha is a youth resilience approach that seeks to facilitate development of psycho-social assets to promote positive youth development.  2) Peer facilitators were young women aged 20–30 years. They were each assigned to eight groups with 12–15 participants per group that met weekly for 15 consecutive weeks. | Facilitators received 5 days of training covering group facilitation skills and curriculum content, followed by a 2-day refresher training after completing eight modules. A team leader assisted facilitators with planning and reporting, and filled in if a peer facilitator was unavailable. |
| Mathias 2019  94; High | India | **Qualitative**  Grounded theory (interviews and FGDs) | Intervention participants (n=112)  Peer facilitators (n=8)  Young people living in four target communities, aged under 25 years, self-reporting that they experienced mental health problems. | Facilitator  Peers were mixed-sex young people belonging to the local community, aged under 30 years, with personal experience of mental ill-health and who had completed 12 class in high school. | **Community-based; Psychosocial**  Peer-facilitated weekly group sessions to promote youth development and resilience and reduce distress | 1) Eight peer facilitators were selected from the local community, who had experience of mental health (as a caregiver or personally).  2) The participants were young people, under 25 years, with self-reported mental health problems (from non-specific distress to depression or addiction).  3) The facilitators led 17 interactive sessions over five months, using the 'Nae Disha' curriculum, aimed at youth development and mental health promotion. The curriculum focused on character strengths, emotion management, and planning, emphasized building emotional resilience through participatory activities.  4) Participants were encouraged by the facilitators to participate in collective activities such as the preparations during the International Youth Day. | Supervision and refresher courses were provided by the project staff to the facilitators. |
| Nasution 2019  85; High | Indonesia | **Quantitative**  RCT | Total participants (n=86)  TKN + CBT + PL group (n=43)  TKN only (n=43)  Adolescents from class XI, senior high school. | Leaders  Peer definition not reported. | **School-Based; Counselling**  Combination of training by mental health nurses (TKN), CBT and Peer leadership to reduce suicidal ideation in adolescents | 1) Intervention group 1 received training by mental health nurses (TKN), CBT, and PL, while Intervention group 2 received only TKN.  2) implementation of Peer leadership actions aimed at changing participants’ behaviour | N/A |
| Ndongo 2025  59; Moderate | Cameroon | **Quantitative**  RCT | Intervention group (n=126)  Control group (n=152)  Adolescents aged 10-19 who were living with vertically acquired HIV, receiving cART, on follow-up at the clinic. | Facilitators  Peers were adolescents living with HIV. | **Community-based; Support group**  Peer-facilitated support groups | 15-month “KidAid Cameroun” intervention consisting of family-level interventions including support groups for ALHIV facilitated by peers, support groups for parents/guardians facilitated by ALHIV peer supporters, and leisure groups supervised by artists. | Not reported |
| Rodriguez 2021  85; High | China | **Quantitative**  RCT | Total (n=54)  MIND+ group (n=27)  MIND (control) group (n=27)  Peer counsellors (n=4)  University students (mean age 23.5) who were proficient in English, and not currently receiving formal mental health treatment. | Counsellor  Peers were currently enrolled university students in Beijing, who had smartphone or internet access, ability to read and communicate in Mandarin and English. | **School-based; Counselling**  Internet-Based mindfulness intervention plus peer counsellor support (MIND+) | 1) The web-based component delivered mindfulness-based cognitive therapy in an internet course that can be completed in 4 weeks and included 4 videos, audio files, assignments, and exercises  2) Throughout the course of therapy, peer counsellors were directed to conduct brief (15–20 minute) weekly phone or text talks with MIND+ participants in order to help and encourage them to complete the web-based intervention. | Peer counselors received 8-hour in-person training prior to the trial and attended supervision meetings throughout the study |
| Simms 2022  Quantitative: 73; Moderate  Qualitative:  94; High | Zimbabwe | **Quantitative + Qualitative**  Cluster randomized trial + qualitative component (case reviews, FGDs and audio diaries) | Zvandiri-PST arm:  Baseline (n= 421), 48-weeks Follow-up (n= 377)  Zvandiri arm:  Baseline (n= 421), 48-weeks Follow-up (n= 388)  Interviewed CATS (n=20)  Adolescents living with HIV aged 10-19, who were taking ART and had SSQ score 7+. | Counsellor  Peers were undergraduate students of mixed sexes studying psychology and serving a 10-month attachment with the Friendship Bench. | **Community-based; Counselling**  Problem-solving therapy offered by trained peers (CATS) through counselling, and support groups (Zvandiri-PST) | 1) Community Adolescent Treatment Supporters (CATS) are HIV-positive young people aged 18 to 24 who are trained and monitored to provide peer-counselling and support.  2) Problem-solving therapy (PST) is a cognitive behavioural approach that consists of a series of activities which develop cognitive tools for problem-solving as well as adaptive skills and a stronger sense of agency. | CATS received training to provide counselling and support.  CATS in the Zvandiri-PST arm met with a Zvandiri mentor at least once every two weeks to discuss individual cases |
| Tinago 2024  56; Moderate | Zimbabwe | **Quantitative**  Pre-post with control | Intervention group (n=104)  Control group (n=79)  Peer educators (n=12)  Adolescent mothers aged 15-18 who were pregnant and/or had a child or children, residing in the two low-income high-density communities. | Facilitators  Peers were aged between 19 and 25 years, who had given birth during adolescence and had at least 7^th^ grade education. | **Community-based; Support group**  Peer support group intervention to mitigate social isolation and stigma of adolescent motherhood | 1) Peer support groups met in-person twice a month and completed 12 total 75-min peer-group sessions.  2) WhatsApp was used by participants and facilitators to schedule meetings, ask and respond to questions, and further discuss peer-support group topics. | Community health workers and peer educators were trained on implementing the curriculum by the project coordinator and local subject matter experts during a 3-day training session |
| Vancampfort 2025  41; Low | Uganda | **Quantitative**  Cluster randomized trial | Intervention group (n=2598)  Control group (n=1303)  Students aged 14-17. | Facilitators  Peers were volunteer students in the class who had lived experience related to the topics discussed, and had leadership, empathy and communication skills. | **School-based; Psychosocial**  Act-belong-commit-based (ACT) group intervention | 1) 12, 2 hour sessions including 30 min physical activity and 1.5 hours of group based discussions and reflections on mental health topics (ACT intervention).  2) First 6 sessions facilitated by the teacher and final 6 sessions were facilitated by volunteer-peer facilitators who had lived experience of topics that were discussed.  3) Peer-led sessions repeated content of first 6 teacher-led sessions. | Teachers received training from a psychologist during a 2-day, 10hr workshop.  Peer training not reported. |
| Wambua 2019  59; Moderate | Kenya | **Qualitative**  Action research (series of group meetings) | Peer mentors (n=9)  Adolescents living with HIV aged 10-24. | Mentors  Peers were young adults and older individuals between late 30s and 40s who entered the facility as adolescents seeking care but had gathered extensive experience in working with adolescents. | **Community-based; Counselling**  Peer-led psychotherapy intervention for HIV-infected adolescents | 1) The intervention was led by peer mentors who specialized in working with young participants, specifically those living with HIV (YLHIV).  2) The intervention team also included two nurses, two clinical officers, and one pharmacist. These professionals provided technical expertise and their experience in working with YLHIV.  3) Nine peer mentors from an outpatient Comprehensive Care Centre (CCC) were selected for the program. These individuals were chosen because of their status as "health champions," characterized by their low viral load and good adherence to medication.  4) The program placed a special emphasis on various competencies, such as managing self-esteem issues, addressing self-stigma and discrimination, promoting healthy eating and positive living, building a strong social support network, and enhancing self-esteem and confidence. | Peer mentors received training to address both short-term and long-term challenges related to HIV. This training included information on HIV-related issues and community resources available for young people.  The sessions aimed at enhancing the mentors' skills in communication, listening, and attending. They also focused on increasing their knowledge of mental health issues intersecting with HIV/AIDS. |
| Wogrin 2019  94; High | Zimbabwe | **Qualitative**  Action research (FGDs) | Adolescents (n = 4), Peer grief counsellors (n = 1), Total intervention participants (n= 330)  Adolescents living with HIV aged 10-22 | Counsellor  Peers were volunteers who helped develop the intervention, aged 18-21 years and who had contracted HIV perinatally and experienced multiple losses. | **Community-based; Counselling**  Peer-led bereavement group-based intervention | 1) The intervention was facilitated by 10 specially trained peer grief counselors (PGCs). 2) The intervention consisted of six sessions, integrated within existing community-based HIV support groups for adolescents. 3) Caregivers of the adolescents participating in the HIV support groups were also involved, receiving one session during a regular quarterly support group meeting. 4) The group session topics are as follows:  -Setting the stage, defining grief  -Personal stories  -Exploring feelings  -Feelings and coping strategies  -Coping strategies  -Commemoration and closing 5) The size of the groups varied, ranging from 9 to 58 participants, reflecting the diverse characteristics of different communities. The majority of the participants in these groups were adolescents, ranging in age from 10 to 22 years old. | The PGCs received supervision, where both the content delivery and the process of the group sessions were reviewed, and feedback was provided. |
| Wogrin 2021  94; High | Zimbabwe | **Qualitative**  Case reviews and FGDs | CATS (n=20)  Participants are the peers. | Counsellor  Peers were aged 18-23, HIV positive, virally suppressed, able to read and write, psychologically stable and able to serve as a role model. | **Community-based; Counselling**  Problem-solving therapy offered by trained peers (CATS) | 1) Community Adolescent Treatment Supporters (CATS) are HIV-positive young people aged 18 to 24 who are trained and monitored to provide peer-counselling and support.  2) Problem-solving therapy (PST) is a cognitive-behavioural approach that consists of a series of activities which develop cognitive tools for problem-solving as well as adaptive skills and a stronger sense of agency. | All CATS underwent a standard initial 5-day training focused on understanding HIV and its treatment, including the psychosocial aspects of living with HIV, basic counseling techniques, and support strategies. |

# **Table S4. Summary of study findings**

| **Study ID** | **Approach and methods** | **Participants** | **Intervention Type** | **Relevant Outcomes/ Phenomena of interest** | **SMD and CI** | **Qualitative Findings** |
| --- | --- | --- | --- | --- | --- | --- |
| Balaji 2011 | **Quantitative**  Cluster randomized trial | Rural:  Baseline(n= 1803)  Follow-up(n= 1620)  Urban: Baseline(n=1860)  Follow-up(n= 1942) | **Community/School-based;**  **Psychoeducation**  Yuva Mitr (friend of youth): a multicomponent (peer/teacher) health education intervention | Substance use (%) Suicidal behaviour (%) Probable depression (%)  Help seeking for stress concerns (%)  Knowledge and attitudes about emotional health (%) Knowledge and attitudes about substance use (%) | Sample sizes were not specified for intervention and comparison arms | N/A |
| Beji-Chauke 2025 | **Quantitative + Qualitative** Cluster randomized trial + qualitative component (interviews) | Interview (n=13)  Intervention group (n=187)  Control group (n=86) | **Community-based;**  **Counselling**  YouFB: Youth Friendship Bench group counselling led by peers | Mean score of common mental health disorders (SSQ-14)  Prevalence (%) of common mental health disorders (SSQ-14)  Mean score of depression (PHQ-9)  Prevalence (%) of depression (PHQ-9)  Mean score of anxiety (GAD-7)  Prevalence (%) of anxiety (GAD-7)  Mean score of WHO-Disability Assessment Schedule (WHO-DAS)  Prevalence (%) of WHOS-Disability Assessment Schedule (WHO-DAS)  Participants’ perceived feasibility and acceptability of intervention (Qualitative)  Peer supporters’ perceived feasibility of intervention (Qualitative) | SSQ: d= -0.2565, 95%CI= (-0.5128, -0.0003)  SSQ >=8: d= -0.2712, 95%CI= (-0.5569, 0.0144) PHQ-9: d= -0.4076, 95%CI= (-0.6653, -0.15) PHQ-9 >=11: d= -0.4123, 95%CI= (-0.7456, -0.079) GAD-7: d=0.022, 95%CI= (-0.2334, 0.2774)  GAD-7 >=10: d=0.0664, 95%CI= (-0.2321, 0.365)  WHO-DAS: d=0, 95%CI= (-0.2554, 0.2554)  WHO-DAS >=20: d=0.0733, 95%CI= (0.1684, 0.3732) | Participants:  1) High acceptability of peer-delivered support: preference of YouFB buddies over adult CHWs, described as more empathetic, relatable and better able to understand youth-specific concerns without judgement  2) Importance of age and gender matching: increased comfort, openness, and willingness to discuss sensitive topics  3) Safe spaces and empowerment: increased confidence, improved problem-solving, strengthened relationships  4) Positive psychosocial and practical impacts: improved emotional well-being, interpersonal relationships, substance use, and income-generating activities  Peers:  1) Benefits for peers: personal growth, increased confidence, practical skill development, greater clarity about career goals  2) Need for supervision and emotional support  3) Limits of peer support and structural barriers: many challenges cannot be addressed through only counselling  5) Implementation challenges: disruptions by external factors (COVID-19), time constraints, broader socioeconomic barriers |
| Bhatia 2023 | **Quantitative**  Cluster randomized trial | Intervention group (n=737)  Control group (n=741)  Peer educators (n=30) | **Community-based;**  **Psychoeducation**  Peer education on nutrition, health, mental health and violence | Mean score of common mental health disorders (Brief Problem Monitor-Youth)  Mean score of self-efficacy (The Schwarzer’s General Self-Efficacy Scale)  Mean score of resilience (Child and youth Resilience Measure 11-item) | BPM-Y: d= 0.1008, 95%CI= (=0.0012, 0.2029)  GSE: d= 0.2708 , 95%CI= (0.1684, 0.3732)  CYRM: d= 0.3323, 95%CI=(0.2296, 0.4349) | N/A |
| Chingono 2022 | **Quantitative + Qualitative**  Pre-post without control + qualitative component (interviews and FGDs) | Interview (n=18)  Baseline survey (n= 93)  Follow-up survey (n=51) | **Community-based;**  **Support group**  Self-help, peer education and practice groups | Prevalence (%) of common mental health disorders (SSQ-14)  Perceived practical and emotional support from peers (Qualitative)  Struggles with life stressors (Qualitative) | SSQ-14: d= -1.2314, 95% CI= (-2.3546, -0.1083) | Four themes emerged from the focus group discussions:  1) Struggles with life stressors  Interviewees described how stressful life events contributed to emotions of despair and anxiety. Some were forced to leave home at a young age due to abuse in the home, and they began selling sex to survive. Others said that their lives were turned upside down when they found themselves in a relationship with verbal, emotional, and/or physical abuse.  2) Burden of motherhood  Failure in one area of personal life harmed AGYW's confidence in their capacity to cope with becoming young moms.  3) Social stigma and Isolation related to sex work  Involvement in selling sex deteriorated adolescent girls and young women (AGYW) self-esteem, particularly in the face of stigma in the community, which was exacerbated by their status as single moms.  4) Self-help groups as a source of hope  Joining a SHG provided an opportunity to interact with peers and pleasant, nonjudgmental staff in a socially supportive environment. Participants with minimal formal schooling experience saw the intervention as an alternative learning environment and enrolled to gain new knowledge and skills. |
| Chory 2022 | **Quantitative + Qualitative**  Pre-post without control + qualitative component (pre-post interviews) | Baseline (n=30) Follow-up (n=29)  Pre-intervention interview  (n=25) Post-intervention interview  (n=15) | **Community-based;**  **Support group**  Peer group discussions on social media (WhatsApp) monitored by a counsellor | Prevalence (%) of Depression (PHQ-9)  Prevalence (%) of Depression (Hopkins)  Prevalence (%) of Anxiety (Hopkins)  Mean Social and behavioural difficulties (SDQ)  Participants’ experiences, acceptability and feasibility of mental and behavioural health counselling and peer support through WhatsApp® (Qualitative) | PHQ-9: d= 0.0336, 95% CI= (-1.5329, 1.6001)  Hopkins-depression: d= 0.0336, 95% CI= (-1.5329, 1.6001)  Hopkins-anxiety: d> 10, 95% CI= (-inf, +inf)  SDQ: d= 0.3644, 95% CI= (-0.1502, 0.8791) | Mental Health Knowledge: Around 60% had some awareness but no personal experiences with mental health issues or services.  Perceptions of Mental Illness: Described as "madness," "crazy," or "sickness," often linked to major life adversities.  Understanding of Depression: Associated with suicidal thoughts, self-destructive behaviours, anger, stress, unhappiness, and aggression.  Awareness of Anxiety: Better understood, characterized as fear and restlessness.  Personal Connections: Only 20% knew someone with depression or anxiety.  Value of WhatsApp Chats: Provided community and peer support among adolescents living with HIV (ALWH).  Barriers to Peer Support: Lack of interaction with other ALWH.  Challenges to Participation: household/school commitments, power outages, limited phone credit, internet issues, and learning to use mobile devices.  Caregiver Influence: Some caregivers faced barriers due to misunderstanding the use of phones.  Preferred Discussion Topics: Hope, stress, stigma, mental health/HIV myths, and treatment adherence.  Program Expansion: Consensus on expanding the mobile-based counselling and peer support program.  In-person Meetings Suggestion: Use school holidays to arrange meetings with ALWH and counsellors.  Future Chat Topics: Relationships, Conflict management, and Disclosure/Confidentiality issues specific to ALWH. |
| Dhand 2006 | **Qualitative**  Ethnography (participant observation and semi-structured interviews) | Peer educators (n=8) | **Community-based;**  **Psychoeducation**  Peer education outreach program targeting heroin addicts | Interaction patterns observed in outreach role-plays | N/A | Three main interaction patterns were identified:  1) Counsellor-client: focusing on building rapport, understanding the addict's situation, and co-planning future steps;  2) Doctor-patient: educators explained illnesses, suggested treatments, and occasionally admonished clients for lack of caution, especially with ill clients;  3) Role model-follower: educators shared personal experiences and offered motivational mentoring |
| Ding 2020 | **Quantitative**  RCT | Intervention group (n=70)  Control group (n=71) | **School-based;**  **Psychoeducation**  Peer education on health knowledge and behaviours concerning COVID-19 AND Physical exercise program (Aerobics) | Mean Anxiety (SAS)  Mean Depression (SDS)  Mean Sleep quality (PSQI) | SAS: d= -0.9677, 95% CI=  (-1.3166, 0.6188  SDS: d= -0.5685, 95% CI=  (-0.9052, 0.2318)  PSQI: d= -1.0323, 95% CI=  (-1.3837, -0.6809) | N/A |
| Dow 2018 | **Quantitative + Qualitative**  RCT + Qualitative feasibility study (session notes and interviews) | Intervention group (n=58) | **Community-based; Counselling**  Group counselling led by peers | Feasibility (attendance and fidelity to the intervention)  Acceptability (Qualitative)  a. Stress and worries  b. Resilience and ways of coping with stress | Youth attendance: Wave 1= 77%, Wave 2 = 85%, Wave 3 = 92%  Caregivers attendance: Wave 1 = 13%; Wave 2 = 63%; Wave 3 = 69% | For acceptability, there were themes generated from the weekly group sessions:  1) Identifying and coping with stressful events.  2) Promoting strong familial and social relationships.  3) Creating a safe and healthy living environment by improving disclosure skills, identifying and realizing one’s own life values, and reducing stigma related to the issues discussed in the sessions. |
| Eryılmaz 2017 | **Quantitative + Qualitative**  Pre-post with control group + qualitative component (follow-up interviews) | Total (n= 60):  Experiment group (n=30),  Control group (n=30) | **School-based; Counselling**  Peer-helping counselling program | Mean Satisfaction with life (SWF)  Mean Positive affect (PANAS-PA)  Mean Negative affect (PANAS-NA)  Participants' opinions and feelings about the program (Qualitative)  Helpers' opinions and feelings about the program (Qualitative) | SWF: d= 0.9272, 95% CI= (0.3947, 1.4598)  PA: d= 1.4915, 95% CI= (0.9194, 2.0636)  NA: d= -0.8355, 95% CI= (-1.3631, -0.3078) | Regarding the effectiveness of the intervention in terms of well-being, the participants indicated the following:  1) Increased happiness  2) Increased awareness  3) Supported feelings of strength  4) Increased optimism  5) Increased self-regulation  6) Fostered positive relationships  The helpers’ evaluations regarding the intervention included:  1) Benefits to the participants  2) Increased competence  3) Increased happiness  4) Increased awareness  5) Fostered positive relationships |
| Ferris France 2023 | **Qualitative**  Qualitative evaluation study (individual interviews and focus group discussions) | Intervention participants (n=62)  Peer coaches (n=15) | **Community-based;**  **Support group**  Peer-delivered inquiry-based stress reduction (IBSR) support groups | Perceived impact of the *Wakakosha* intervention | N/A | Positive changes were identified among intervention participants including the following:  1) Self-confidence  2) Self-agency  3) Sense of purpose/meaning  4) Body positivity  5) Improved communication and personal/family relationships  6) Forgiveness  Practical skills were also gained through this intervention, including self-inquiry, mindfulness, meditation and creativity. |
| Fuhr 2019 | **Quantitative**  RCT | Intervention participants:  Baseline (n=140), 3-month follow-up (n=123), 6-month (n=122)  Control participants:  Baseline (n=140), 3-month follow-up (n=122), 6-month (n=129) | **Community-based;**  **Counselling**  Peer-delivered behavioural activation sessions (Thinking Healthy Programme Peer-delivered (THPP)) | Mean PHQ-9 score for depressive symptoms at 6 months  Mean PHQ-9 score for depressive symptoms 3 months  Remission (%) at 6 months  Remission (%) at 3 months  Recovery (%)  Mean WHO-Disability Assessment Schedule score (WHO-DAS) at 6 months  Mean WHO-Disability Assessment Schedule score (WHO-DAS) at 3 months | PHQ-9 at 6 mo: d= -0.2096, 95%CI= (-0.4578, 0.0386)  PHQ-9 at 3 mo: d= -0.3076, 95%CI= (-0.5595, -0.0557)  Remission at 6 mo: d= 0.3248, 95%CI= (0.0311, 0.6185)  Remission at 3 mo: d= 0.2015, 95%CI= (-0.0775, 0.4805)  Recovery: d= 0.2669, 95%CI= (-0.0171, 0.5509)  WHO-DAS at 6 mo: d= -0.2058, 95%CI= (-0.4544, 0.0429)  WHO-DAS at 3 mo: d= -0.2988, 95%CI= (-0.5507, -0.047) | N/A |
| Garriott 2023 | **Qualitative**  Qualitative evaluation study (semi-structured interviews) | Intervention participants (n=22)  Peer counsellors (n=18) | **Community-based;**  **Counselling**  Problem-solving therapy (PST) peer counselling intervention | Attitudes and impressions of YLWH of Safe Haven  1) Initial expectations  2) Development of therapeutic relationships with peers  3) Challenges faced with peers  4) Overall impressions of the intervention  5) Advice to future participants of peer counselling | N/A | Initial expectations:  1) Fear and apprehensions before attending sessions  2) Openness to receiving help  The peer counsellor relationship: 1) They were easy to relate to  2) Approachable and friendly  3) Feeling and knowing there are resources for support  4)instilled confidence  Challenges with the peer counsellor relationship:  1) Coming in late  2) Finishing off very quickly  3) Not very interesting sessions  Overall impressions of the Safe Haven intervention  1) Freedom of expression  2) Receiving practical advice  3) professional and well-trained peer counsellors  Advice to future participants:  1) Peer counsellors want to and can support youth  2) Importance of a nonjudgemental stance when seeking counselling |
| Gayathri 2024 | **Quantitative**  RCT | Intervention group(n=30)  Control group (n=30) | **Hospital-based;**  **Psychosocial**  Peer-assisted therapy | Mean score on Social Phobia Scale (SPS)  Mean score on Social Interaction Anxiety Scale (SIAS) | SPS: d= -0.2992, 95%CI= (-0.8082, 0.2098)  SIAS: d= -0.4776, 95%CI= (-0.9911, 0.0359) | N/A |
| Harrison 2023 | **Quantitative + Qualitative**  Post-intervention with control + qualitative component (in-depth interviews) | Intervention group (n=20)  Control group (n=38)  Post-intervention interview (n=20) | **Hospital-based; Support group**  Peer-led group support session | Mean resilience (CD-RISC)  Mean score on Child Attitude Toward Illness Scale (CATIS)  Mean score Berger Scale of Disease-Related Stigma  Mean score on HIV-stigma scale (ALHIV-SS)  Mean self-concept (Beck scale)  Mean anxiety (Beck scale)  Mean depression (Beck scale)  Mean anger (Beck scale)  Mean disruptive (Beck scale)  Adolescents’ acceptability and willingness to participate in the program (Qualitative) | CD-RISC: d= 0.822, 95%CI (0.2602, 1.3837)  CATIS: d= 1.0146, 95%CI (0.4425, 1.5867)  Berger: d= -0.792 , 95%CI (-1.352, -0.2317)  ALHIV-SS: d= -0.3813, 95%CI (-0.9272, 0.1646)  Self-concept: d= 0.5841, 95%CI (0.0323, 1.1359)  Anxiety: d= -0.2465, 95%CI (-0.7898, 0.2968)  Depression: d= -0.5085, 95%CI (-1.0578, 0.0408)  Anger: d= -0.2904, 95%CI (-0.8344, 0.2536)  Disruptive: d= -0.2274, 95%CI (-0.7704, 0.3157) | 1) Meeting young people with different illnesses was eye-opening and powerful: sharing common experience of living with chronic illness, combatting feelings of isolation  2) Finding support and acceptance in the support group: safe space to freely express themselves and to be understood  3) Benefits of participation in the support group |
| Im 2018 | **Quantitative**  Pre-post without control | Intervention participants (n=145)  Survey participants (n=141)  Moderate to severe PTSD (n=45)  None to low PTSD (n=96) | **Community-based;**  **Psychoeducation**  Peer-led Trauma-Informed Psychoeducation (TIPE) | Mean PTSD symptoms (PTSD Check List – Civilian Version (PCL-C))  Mean PTSD symptoms (PTSD Check List – Civilian Version (PCL-C)), Low  Mean PTSD symptoms (PTSD Check List – Civilian Version (PCL-C)), High | PCL-C total: d= -0.0765, 95% CI= (-0.3101, 0.157)  PCL-C Low: d= 0.6905, 95% CI= (0.3993, 0.9817)  PCL-C High: d= -1.6287, 95% CI= (-2.1055, -1.1519) | N/A |
| Jaguga 2023 | **Qualitative**  Qualitative evaluation study (focus group discussions and semi-structured interviews) | Intervention participants (n=100)  Focus group participants (n=25)  Peer providers (n=4) | **Clinic-based; Counselling**  Peer-provided Screening and Brief Intervention program | Participants’ perceptions on the acceptability of the program  Peer providers’ and clinic leaders’ perceptions on the feasibility and acceptability of the program | N/A | Participants’ feedback on the program:  1) Program content and delivery: Content was relatable  2)Perceptions of the peer providers: Able to trust; comfortable due to shared experiences and similar age; friendly, open and skilled  3) Impact on youth behaviour: Steps taken to quit or cut down on substance use  4) Youths’ perceptions of usefulness of intervention: Useful information about harmful effects of substance use  5) Recommendations for improving the program: Translation to Swahili or other local languages, increased length and number of sessions, for peer providers to share their experiences  Peer providers’ perceptions of the program:  1) Confidence in administering the program  2) Program is compatible with the goal and mission of the clinic  3)Program adequately meets the needs of youth at the clinic |
| Jaguga 2025 | **Qualitative**  Qualitative pilot study (individual semi-structured interviews) | Intervention participants (n=38)  Interview participants (n=25)  Peer provider (n=1) | **Clinic-based; Counselling**  Peer-provided Screening and Brief Intervention | Participants’ perspective on the acceptability of intervention | N/A | 1) Affective attitude: Youth enjoyed the session content and interacting with the peer provider  2) Burden: Youth found understanding and participating in the session easy  3) Perceived effectiveness: Youth perceived the intervention to be effective in reducing substance use and improving their well-being  4) Ethicality: The counselling session was a good fit with their goals and values  5) Intervention coherence: The overall goal of the intervention was well understood.  6) Opportunity costs: Intervention attendance interfered with other activities, such as work, school, sports, gaming, visiting family, house chores  7) Self-efficacy: Youth were confident about their ability to reduce or stop using substances |
| Kavya 2020 | **Quantitative**  Post- intervention with control (semi-structured questionnaire) | Bihar (n=345):  Participants (n= 180),  Non-participants (n= 165) Jharkhand (n = 406):  Participants (n= 315),  Non-participants (n=91)  Karnataka (n=267):  Participants (n=186),  Non-participants (n=81)  Maharashtra (n=308):  Participants (n=287),  Non-participants (n=21) | **Community-based; Psychoeducation**  Peer-led intervention on knowledge, attitude and practices (KAP) related to sexual reproductive and mental health issues | Less worry (%)  Less restless (%)  Feeling lonely (%)  Consumption of tobacco and alcohol (%) | Less worry a: d= 0.15, 95%CI= (-0.1256, 0.4257)  Less worry b: d= -0.1354, 95%CI= (-0.469, 0.1982)  Less worry c: d= -0.1181, 95%CI= (-0.4904, 0.2542)  Less worry d: d= -0.529, 95%CI= (-0.9418, -0.1163)  Less restless a: d= -0.048, 95%CI= (-0.4068, 0.3108)  Less restless b: d= -0.1206, 95%CI= (-0.4561, 0.2149)  Less restless c: d= -0.2239, 95%CI= (-0.6193, 0.1716)  Less restless d: d= -0.2894, 95%CI= (-0.7715, 0.1927)  Feeling lonely a: d= -0.1711, 95%CI= (-0.4739, 0.1317)  Feeling lonely b: d= 0.5893, 95%CI= (0.1842, 0.9945)  Feeling lonely c: d= -0.5791, 95%CI= (-0.878, -0.2803)  Feeling lonely d: d= -0.0855, 95%CI= (-0.5769, 0.4059)  tobacco and alcohol a: d= -0.325, 95%CI= (-0.827, 0.1771)  tobacco and alcohol b: d= 0.1426, 95%CI= (-0.4772, 0.7623)  tobacco and alcohol c: d= 0.2765, 95%CI= (-0.4147, 0.9677)  tobacco and alcohol d: d≃ 10, 95%CI= (-inf, +inf)  *a= Bihar b= Jharkhand c= Karnataka d= Maharashtra | N/A |
| Kermode 2021 | **Quantitative**  Pre-post without control | Intervention participants (n=142) | **Community-based; Support group**  Peer-facilitated, participatory intervention for young people affected by mental illness | Social participation (Social participation scale)  Common mental health problems (GHQ-12)  Mental health problems and emotional and behavioural strengths of children and adolescents (Strengths and difficulties questionnaire(S&D)) | SDs were not reported. | N/A |
| Keyan 2025 | **Quantitative**  RCT | Coping with COVID (n=91)  Supportive Counselling (n=92) | **School-based; Psychoeducation**  Peer-led psychoeducation program | Mean depression (HADS)  Mean anxiety (HADS)  Mean anxiety (GAD-7)  Mean score on COVID stress scale  Mean wellbeing (WHO-5)  Mean suicidality (SIDAS) | HADS depression: d= -0.0081, 95%CI= (-0.2978, 0.2817)  HADS anxiety: d= -0.0665, 95%CI= (-0.3564, 0.2234)  GAD-7: d= -0.0152, 95%CI= (-0.3050, 0.2746)  COVID stress: d= -0.2362, 95%CI= (-0.5270, 0.0546)  WHO-5: d= 0.1074, 95%CI= (-0.3974, 0.3862)  SIDAS: d= 0.0962, 95%CI= (-0.1937, 0.3862) | N/A |
| Li 2024 | **Quantitative**  RCT | Intervention group (n=45)  Control group (n=45)  Peer supporters (n=5) | **Hospital-based; Support group**  Peer-facilitated group discussion sessions | Mean score of psychological distress (Distress thermometer (DT))  Mean anxiety (HADS)  Mean depression (HADS)  Mean score of perceived peer support (CaPSS) | DT: d= -2.0965, 95%CI= (-2.6129, -1.5801)  HADS anxiety: d= -0.2648, 95%CI= (-0.6799, 0.1502)  HADS depression: d= -0.5648, 95%CI= (-0.9864, -0.1433)  CaPSS: d= 0.3377, 95%CI= (-0.0785, 0.7539) | N/A |
| Machisa 2023 | **Quantitative + Qualitative**  pre-post without control + qualitative component (semi-structured interviews) | Intervention participants (n=98)  Interview participants (n=35) | **School-based; Psychosocial**  Peer-facilitated group discussion sessions | Mean self-esteem (Rosenberg’s scale)  Mean depressive symptoms  Participant reported outcomes after intervention (Qualitative) | Self-esteem: d= 0.2421, 95%CI= (-0.0389, 0.5232)  Depressive symptoms: d= -0.4359, 95%CI= (-0.7192, -0.1526) | 1) Confidence to verbally and physically resist against non-partner harassment  2) Confidence to use physical self-defence tactics with intimate partners  3) Improved assertive communication, negotiation skills and shifts in intimate relationship dynamics  4) Shifts in gender beliefs and exiting abusive relationships  5) Improved vigilance and strategies to minimize sexual assault risks |
| Maselko 2020 | **Quantitative**  Cluster randomized trial | Intervention group:  Baseline (n=283)  36-months follow-up (n=206)  Control group:  Baseline (n=287)  36-months follow-up (n=216) | **Community-based; Psychosocial**  Peer-delivered psychosocial intervention (THPP+) including peer-support, behavioural activation, and problem-solving | Mean Maternal Depressive symptoms (PHQ-9)  Prevalence (%) of Remission rate (PHQ-9 < 10 score)  Prevalence (%) of Maternal current major depressive episode (SCID)  Mean Maternal Disability score (WHO-DAS) | PHQ-9: d= -0.1061, 95% CI= (-0.2971, 0.085)  PHQ-9 <10: d= 0.0059, 95% CI= (-0.2375, 0.2493)  SCID: d= -0.2026, 95% CI= (-0.4717, 0.0666)  WHO-DAS d= -0.1035, 95% CI= (-0.2945, 0.0875) | N/A |
| Mathias 2018 | **Quantitative**  Pre-post without control | Intervention participants (n=106) | **Community-based; Psychosocial**  Peer-facilitated weekly group sessions to promote youth development and resilience and reduce distress | Mean Coping ability and resilience (Connor-Davidson resilience scale (CD-RISC))  Mean Depression, anxiety and optimism (Schwarzer’s General Self-Efficacy Scale)  Mean Depressive symptoms score (PHQ-9)  Mean Anxiety symptoms score (GAD-7) | CD-RISC: d= 10.0019, 95% CI= (9.0126, 10.9913)  SGSES: d= 7.2276, 95% CI= (6.2892, 7.728)  PHQ-9: d= -8.2824, 95% CI= (-9.1154, -7.4493)  GAD-7: d= 5.5131, 95% CI= (4.9233, 6.1029) | N/A |
| Mathias 2019 | **Qualitative**  Grounded theory (interviews and FGDs) | Intervention participants (n=112) | **Community-based; Psychosocial**  Peer-facilitated weekly group sessions to promote youth development and resilience and reduce distress | To assess the impact of a peer-led, community-based, participatory group intervention on social inclusion and mental health:  Participants' experience  Peer facilitators' experience | N/A | The intervention yielded the following outcomes:  1) formation of new peer friendship networks 2) increased self-efficacy 3) improved mental health 4) increased community participation  Outcomes specific to young women:  1) increased freedom of movement 2) greater confidence in communicating  Outcome specific to young men:  1) Changes in community perceptions of the young men  Contextual and intervention factors influencing outcomes:  1) Support from parents 2) skills of peer facilitators 3) limited freedom of movement for young women 4) socioeconomic factors |
| Nasution 2019 | **Quantitative**  RCT | Total participants (n=86)  TKN + CBT + PL group (n=43)  TKN only (n=43) | **School-Based; Counselling**  Combination of training by mental health nurses (TKN), CBT and Peer leadership to reduce suicidal ideation in adolescents | Suicidal ideation (Beck scale) | SDs were not reported. | N/A |
| Ndongo 2025 | **Quantitative**  RCT | Intervention group (n=126)  Control group (n=152) | **Community-based; Support group**  Peer-facilitated support groups | Mean anxiety (Multidimensional anxiety scale for children (MASC))  Mean depression (Child Depression Inventory)  Mean self-esteem (Coopersmith SEI) | MASC: d= -0.5294, 95%CI= (-0.7697, -0.2891)  CDI: d= -0.1429, 95%CI= (-0.3795, 0.0937)  SEI: d= 0.5, 95%CI= (0.2601, 0.7399) | N/A |
| Rodriguez 2021 | **Quantitative**  RCT | Total (n=54)  MIND+ group (n=27)  MIND (control) group (n=27) | **School-based; Counselling**  Internet-Based mindfulness intervention plus peer counsellor support (MIND+) | Mean Depressive symptoms (PHQ-9) Mean Depressive symptoms (DASS-21)  Mean Anxiety symptoms (GAD-7)  Mean Anxiety symptoms (DASS-21)  Mean Stress (DASS-21)  Mean Trait mindfulness (Five-Factor Mindfulness Questionnaire (FFMQ)) | PHQ-9: d= -0.3319, 95%CI= (-0.8689, 0.2052) DASS-21 depression: d= -0.0622, 95%CI= (-0.5958, 0.4713)  GAD-7: d= -0.0722, 95%CI= (-0.6058, 0.4614)  DASS-21 anxiety: d= -0.1111, 95%CI= (-0.645, 0.4227)  DASS-21 stress: d= -0.089, 95%CI= (-0.6227, 0.4447)  FFMQ: d= -0.1827, 95%CI= (-0.7173, 0.3518) | N/A |
| Simms 2022 | **Quantitative + Qualitative**  Cluster randomized trial + qualitative component (case reviews, FGDs and audio diaries) | Zvandiri-PST arm:  Baseline (n= 421), 48-weeks Follow-up (n= 377)  Zvandiri arm:  Baseline (n= 421), 48-weeks Follow-up (n= 388)  Interviewed CATS (n=20) | **Community-based; Counselling**  Problem-solving therapy offered by trained peers (CATS) through counselling, and support groups (Zvandiri-PST) | Mean disorder symptoms (Shona Symptom Questionnaire (SSQ) score)  Prevalence of common mental disorders (SSQ >= 8)  Mean Depressive symptoms (PHQ-9 score)  Prevalence (%) of depression (PHQ-9 >= 10)  Prevalence (%) of poor quality of life (EQ-5D < 1)  CATS’ experiences of provision of support and support needs in real time (Qualitative)  Retrospective reflections of CATS' on their experiences (Qualitative)  Participatory insights into the types of problems participants presented with and the experiences of implementation (Qualitative) | SSQ: d= -0.4425, 95%CI= (-0.5793, -0.3058)  SSQ >=8: d= -0.8496, 95%CI= (-1.2349, -0.4644)  PHQ-9: d= -0.3135, 95%CI= (-0.6058, 0.4614)  PHQ-9 >=10: d= -0.6466, 95%CI= (-1.0113, -0.2818)  EQ-5D <1: d= -0.2359, 95%CI= (-0.3965, -0.0753) | Three key themes emerged through focus group discussions with participants and CATS:  1) Impeded fidelity  (a) Scepticism of the capacity of young people to affect their peers' problems  (b) limited agency of youth to resolve relationally entangled problems  2) Value of peer-led problem-discussion therapy  3) Including others in developing broader mental health support networks  (a) support for client from significant adults  (b) support for CATS |
| Tinago 2024 | **Quantitative**  Pre-post with control | Intervention group (n=104)  Control group (n=79)  Peer educators (n=12) | **Community-based; Support group**  Peer support group intervention to mitigate social isolation and stigma of adolescent motherhood | Mean depression (PHQ-9)  Mean perceived peer support (MSPSS)  Mean score of common mental disorders (SSQ)  Mean score of peer and adult support (PSAS) | PHQ-9: d= -0.8262, 95%CI= (-1.1307, -0.5217)  MSPSS: d= 0.7599, 95%CI= (0.4572, 1.0626)  SSQ: d= -0.7647, 95%CI= (-1.0675, -0.4619)  PSAS: d= 0.8576, 95%CI= (0.5522, 1.1631) | N/A |
| Vancampfort 2025 | **Quantitative**  Cluster randomized trial | Intervention group (n=2598)  Control group (n=1303) | **School-based; Psychosocial**  Act-belong-commit-based (ACT) group intervention | Mean depression (PHQ-9)  Mean anxiety (GAD-7) | PHQ-9: d= -0.4298, 95%CI= (-0.5076, -0.352)  GAD-7: d= -0.4081, 95%CI= (-0.4858, -0.3304) | N/A |
| Wambua 2019 | **Qualitative**  Action research (series of group meetings) | Peer mentors (n=9) | **Community-based; Counselling**  Peer-led psychotherapy intervention for HIV-infected adolescents | Reflection on the process of engagement and preparation of peer mentors in the development of the intervention | N/A | In health facilities, collaborative care and task sharing are innovative ideas, but the process is unusually complicated and lengthy, necessitating researchers, healthcare providers, lay health workers, and key stakeholders to devote attention to and understand each other's concerns. |
| Wogrin 2019 | **Qualitative**  Action research (FGDs) | Adolescents (n = 4), PGCs (n = 1), Total intervention participants (n= 330) | **Community-based; Counselling**  Peer-led bereavement group-based intervention | Feasibility and acceptability of the bereavement intervention | N/A | The following themes emerged from the inquiry:  1) Limited experience in focusing on personal loss and feelings  2) Wide range of emotions identified  3) Relief in learning of shared experiences  4) A need for connection with the deceased  5) An increased ability to identify feelings, connect with their present experience and constructively respond |
| Wogrin 2021 | **Qualitative**  Case reviews and FGDs | CATS (n=20) | **Community-based; Counselling**  Problem-solving therapy offered by trained peers (CATS) | CATS’ experiences of provision of support and support needs in real-time  Retrospective reflections of CATS’ on their experiences | N/A | The following themes emerged as insights from CATS in performing their roles:  1) Strengths of peer supporters, i.e. they consider themselves able to provide the assistance needed by ALHIV  2) Challenges in 'moving-into' mental health peer support  3) Problems faced are complex and relationally embedded  4) I can make it less bad: Revised definition of success   Strategies and support structures for CATS to deliver sustained care  1) Connection with external/government organizations  2) Bringing in caregivers into the process  3) Improving relationship between the caregiver and the adolescent  4) Provision of psychosocial support  5) Provision of sustained supervision to handle difficult situations |
